# Plastic Monolithic Mixed‐Conducting Interlayer for Dendrite‐Free Solid‐State Batteries

**DOI:** 10.1002/advs.202105924

**Published:** 2022-04-28

**Authors:** Bing‐Qing Xiong, Shunqiang Chen, Xuan Luo, Qingshun Nian, Xiaowen Zhan, Chengwei Wang, Xiaodi Ren

**Affiliations:** ^1^ Department of Materials Science and Engineering, School of Chemistry and Materials Science University of Science and Technology of China Anhui 230026 China; ^2^ College of Chemistry & Chemical Engineering Anhui University Hefei 230601 China

**Keywords:** dendrite‐free, lithium aromatic hydrocarbon complex, mixed‐conducting interlayer, solid–solid interface, solid‐state electrolyte

## Abstract

Solid‐state electrolytes (SSEs) hold a critical role in enabling high‐energy‐density and safe rechargeable batteries with Li metal anode. Unfortunately, nonuniform lithium deposition and dendrite penetration due to poor interfacial solid–solid contact are hindering their practical applications. Here, solid‐state lithium naphthalenide (Li‐Naph(s)) is introduced as a plastic monolithic mixed‐conducting interlayer (PMMCI) between the garnet electrolyte and the Li anode via a facile cold process. The thin PMMCI shows a well‐ordered layered crystalline structure with excellent mixed‐conducting capability for both Li^+^ (4.38 × 10^–3 ^S cm^–1^) and delocalized electrons (1.01 × 10^–3^ S cm^–1^). In contrast to previous composite interlayers, this monolithic material enables an intrinsically homogenous electric field and Li^+^ transport at the Li/garnet interface, thus significantly reducing the interfacial resistance and achieving uniform and dendrite‐free Li anode plating/stripping. As a result, Li symmetric cells with the PMMCI‐modified garnet electrolyte show highly stable cycling for 1200 h at 0.2 mA cm^–2^ and 500 h at a high current density of 1 mA cm^–2^. The findings provide a new interface design strategy for solid‐state batteries using monolithic mixed‐conducting interlayers.

## Graphical abstract

Solid‐state organic‐based Li‐Naph(s) interlayer is mechanically soft to form intimate contact with solid electrolyte and Li anode. The 2D *π*‐conjugation structure provides facial transport pathway for delocalized electrons and the large interplanar spacing allows fast Li^+^ diffusion. The uniform Li^+^ flux and electric field at the Li/garnet interface enable dendrite‐free Li nucleation and plating/stripping under high rates.

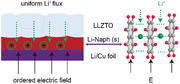

## 1 Introduction

Solid‐state electrolytes (SSEs) are considered promising solutions for high energy density, safe, and long‐life lithium‐metal batteries.^[^ ^1^, ^2^, ^3^, ^4^ ^]^ Especially, Li_7_La_3_Zr_2_O_12_ (LLZO)‐based garnet materials have attracted tremendous attention recently because of their high ionic conductivity, wide electrochemical windows, and excellent stability against Li metal.^[^ ^5^, ^6^, ^7^, ^8^ ^]^ However, the critical obstacle for the deployment of the garnet‐LLZO electrolyte is its poor interfacial contact with Li metal due to its rigid nature and lithiophobic surface^[^ ^9^, ^10^, ^11^, ^12^, ^13^, ^14^ ^]^, which leads to severe cell polarization and dendritic Li deposition due to inhomogeneous current distribution at the solid–solid interface.^[^ ^15^, ^16^, ^17^ ^]^ This effect is also closely related to the quick penetration of Li filaments and failure of solid‐state batteries.^[^ ^18^, ^19^, ^20^, ^21^ ^]^

Although intensive research efforts were taken to modify the Li/garnet interface, the question remains regarding how to achieve ideal interfacial contact and Li^+^ transport for the long‐term cycling stability of Li metal anode in solid‐state batteries. Soft polymer‐based Li^+^‐conducting interlayers (e.g., polyethylene oxide (PEO)) are useful in improving the effective contact area but suffer from Li dendrite growth due to inferior Li^+^ transport issues (≈10^–7^ S cm^–1^ at room temperature).^[^ ^22^ ^]^ Inorganic‐based interlayers, including Li_3_N, Al_2_O_3_, ZnO, Si, Ge, Cu_6_Sn_5_, Li_3_PO_4_, etc., have been used to create lithiophilic interfaces, form Li‐alloys, or facilitate Li^+^ transport.^[^ ^23^, ^24^, ^25^, ^26^, ^27^, ^28^, ^29^, ^30^, ^31^ ^]^ However, rigid inorganic interlayers may lose contact with the garnet or Li anode surface after short cycling due to large volume changes in Li plating/stripping process.^[^ ^32^ ^]^ In addition, despite the higher initial contact area provided by Li‐alloy layers, large interfacial resistances were still frequently encountered, leading to low critical current density (CCD), uneven Li deposition, and limited cycle life.^[^ ^33^, ^34^ ^]^ Previously, composite mixed‐conducting materials (Cu/Li_3_N, Mo/Li_2_S, Li_y_Sn/Li_3_N, etc.) have been investigated as coating materials on garnet electrolytes.^[^ ^35^, ^36^, ^37^ ^]^ However, those composite layers with separated ion‐ (e.g., Li_3_N) and electron‐conducting (e.g., Cu) phases have intrinsic inhomogeneity, which is unfavorable for uniform Li plating and stripping. Furthermore, such bulk heterojunction composite interlayers naturally raise concerns about whether they will facilitate Li growth along the metallic phase during prolonged cycling.

Owing to their good contact with solid electrolytes, liquid‐phase alkali metals aromatic hydrocarbons with solvated electrons have been used as liquid anodes for rechargeable batteries since the 1980s.^[^ ^38^, ^39^, ^40^, ^41^, ^42^ ^]^ However, liquid anodes have very low theoretical specific capacities due to the excessive solvent presence and need an extensive redesign of battery configuration to prevent anode leakage and cell short‐circuiting, which are very challenging for their practical applications.^[^ ^43^ ^]^ Interestingly, we discover that solid‐state lithium naphthalenide (Li‐Naph(s)) possesses excellent mixed‐conducting capabilities for Li^+^ (4.38 × 10^–3^ S cm^–1^) and electrons (1.01 × 10^–3^ S cm^–1^). It can be used as a plastic monolithic mixed‐conducting interlayer (PMMCI) for solid‐state batteries, which effectively addresses the interfacial challenges to enable high rate and long‐term cycling stability of Li anode with garnet electrolyte.

The PMMCI features 1) A layered molecular crystalline structure that enables facial Li^+^ transport across the 2D plane, 2) inherent electrical conductivity with unpaired electrons delocalized in aromatic rings to homogenize the electric field at the interface for uniform Li metal plating/stripping, 3) high chemical stability and excellent compatibilities with both Li anode and the LLZTO solid electrolyte, 4) deformable capability to accommodate volume changes of Li anode during cycling, and 5) low‐cost and simple fabrication process. To the best of our knowledge, this is the first time that the mixed‐conducting properties of Li‐Naph(s) are revealed and used as an interlayer for solid‐state batteries. The PMMCI reduces the Li nucleation energy, promotes the uniform plating of Li metal, and increases the CCD of Li–Li cells to 1.7 mA cm^–2^, thus enabling stable long‐term cycling of solid‐state Li–Li and Li/LiFePO_4_ cells. Our work provides a promising strategy for addressing the poor contact of Li anode with the solid electrolyte and highlights the significance of uniform Li^+^ and e^–^ flux of the interlayer in improving the interfacial charge transfer kinetics for solid‐state batteries.

## 2 Results and Discussion

**Figure 1:**
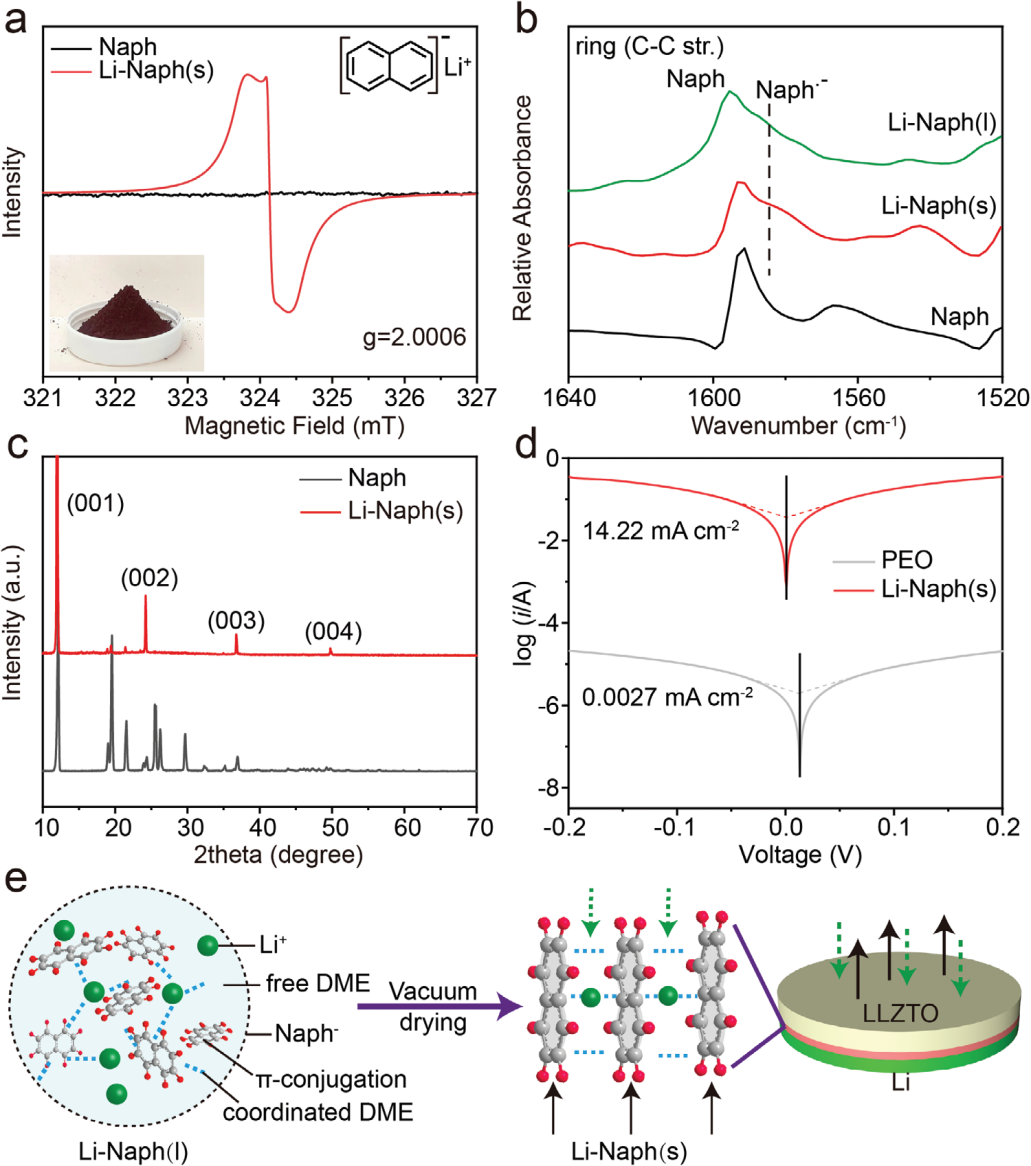
a) EPR spectra of Li‐Naph(s), insets are the corresponding digital image of Li‐Naph(s) powders (bottom) and the molecular formula (top). b) FT‐IR spectra of Li‐Naph(s), Li‐Naph(l) and Naph. c) XRD patterns of Naph and Li‐Naph(s) (minor peaks of Li‐Naph(s) are likely from residual naphthalene molecules). d) Exchange current density measurements of the PEO and Li‐Naph(s) interlayers with Li metal electrodes. e) The schematic diagram for the mixed‐conducting mechanism of the Li‐Naph(s) interlayer.

To understand the effect of the PMMCI on the Li/garnet interface, the physical properties of Li‐Naph(s) were examined. We first prepared Li‐Naph liquid (noted as Li‐Naph(l)) by dissolving lithium metal into a mixed solution of naphthalene and ethers (molar ratio Li: naphthalene = 1:1), which displays a dark green color because of the spontaneous electron donation from Li to the conjugated ring of naphthalene and the formation of stable Naph^–^ radical (photo shown in Figure S1a, Supporting Information). The existence of unpaired electrons delocalized in the *π*‐conjugation ring can be confirmed by the strong electron paramagnetic resonance (EPR) signal shown in Figure S1c–d (Supporting Information). Importantly, even after vacuum drying to remove volatile ether, the resulting solid powders (Li‐Naph(s)) with a reddish‐brown color still show a strong radical signal at *g* = 2.0006 in the EPR test (**Figure** **1**a), which is apparently different from that of Naph. This confirms the preservation of delocalized electrons in Li‐Naph(s), despite the disappearance of hyperfine splitting due to the physical state change. From the FT‐IR spectra (Figure 1b), similar peaks corresponding to C —C aromatic stretching in Naph^–^ radical in Li‐Naph(l) and Li‐Naph(s) were also observed.^[^ ^44^, ^45^ ^]^

In particular, as indicated by the X‐ray diffraction (XRD) pattern in Figure 1c, Li‐Naph(s) has a layered crystalline structure, where the interplanar spacing was calculated to be about 7.39 Å based on the sharp diffraction peak at 2*θ* = 11.96^°^ (*d* spacing estimated to be 7.35, 7.32 and 7.31 Å for the peaks at 24.20°, 36.80°, and 49.85°, respectively, for different orders of reflection). It indicates that naphthalene radical molecules assemble into a layered structure along with ether‐coordinated Li^+^, which remarkably differs from Li‐Naph complexes in the liquid state, as illustrated in Figure 1e. Such configuration should be critical for the excellent mixed‐conducting property of Li‐Naph(s), as it provides an extended *π*‐conjugation system in the monolithic molecular crystalline structure for the transport of delocalized electrons and 2D pathways for the diffusion of Li^+^. At the same time, we found that removing the free DME solvent is essential for the resulting layered structure of Li‐Naph(s). The XRD pattern of Li‐Naph(s) turns back into that of pure naphthalene with the additional DME solvent (Naph: DME = 1:4, molar ratio) as shown in Figure S2d (Supporting Information). Therefore, although DME solvent molecules are important for coordination with Li^+^ to stabilize the Naph^–^ radical anions, their roles are fundamentally different from conventional free‐diffusing solvents, which are the main carriers of ion transport and important for interfacial wetting. The ionic conductivity and electronic conductivity of Li‐Naph(s) are determined to be 4.38 × 10^–3^ and 1.01 × 10^–3 ^S cm^–1^ respectively by the Huggins method (Figure S3 and Table S1, Supporting Information)^[^ ^46^ ^]^, which demonstrates an outstanding mixed‐conducting capability albeit the absence of “free” liquid solvent medium.^[^ ^41^, ^42^ ^]^ To validate the conductivity results from the Huggins method, DC polarization was also carried out to test the electronic conductivity of the Li‐Naph(s). As shown in Figure S4 (Supporting Information), under a DC polarization of 10 mV, the current stabilizes at ≈1.075 mA. After the long‐term DC polarization, the current should be only from electronic conduction with no net flow of Li^+^ ions. The electronic conductivity of the Li‐Naph(s) is calculated to be 1.22 × 10^–3^ S cm^–1^, which is close to the value of 1.01 × 10^–3^ S cm^–1^ obtained from the Huggins method. Because of its unique ion and electron transfer properties, Li‐Naph(s) shows a high exchange current density of 14.22 mA cm^–2^ with Li electrodes, which is several orders of magnitude higher than the conventional PEO interlayer (0.0027 mA cm^–2^, Figure 1d). Despite the portion contributed by electronic conduction, the charge transfer kinetics at the Li/Naph‐Li(s) interface should be significantly improved compared to conventional interlayer materials.

We used Li/Li‐Naph(s)‐garnet/Li symmetric cells to prove the unique function of the PMMCI at the Li/garnet interface with Li_6.4_La_3_Zr_1.4_Ta_0.6_O_12_ (LLZTO) garnet pellets. LLZTO pellets were fabricated according to previous reports (see details in Supporting Information), in which crystal particles are tightly sintered without obvious voids and gaps between the grains, as shown in Figure S5a (Supporting Information). Meanwhile, the XRD pattern of LLZTO as prepared matches well with the standard cubic phase garnet structure (PDF‐045‐0109, Figure S5b, Supporting Information), and the electrochemical impedance spectroscopy (EIS) analysis suggests a high ionic conductivity of 3 × 10^−4^ S cm^–1^ with an activation energy of 0.45 eV (Figure S6, Supporting Information). Li‐Naph(l) was carefully dropped on both surfaces of LLZTO pellet followed by a vacuum‐drying process at room temperature for 15 min to obtain Li‐Naph(s)‐coated LLZTO.

The effect of the Li‐Naph(s) interlayer on the Li anode deposition can be explained by the schematic shown in Figure 1e. The organic‐based Li‐Naph(s) interlayer is mechanically soft to form intimate contacts with both solid electrolyte and Li anode. Owing to its good mixed ionic and electronic conductivity, the thin Li‐Naph(s) interlayer induces uniform Li^+^ flux and electric field at the anode/garnet interface. Note that DME molecules are needed for coordination with Li^+^ to stabilize the Naph^–^ radical anions, and the estimated molar ratio between naphthalene and DME is about 1:2 according to the nuclear magnetic resonance (NMR) of Li‐Naph(s) dissolved in DMSO‐d_6_ (Figure S7, Supporting Information). Scanning electron microscopy (SEM) imaging and energy dispersion spectrum (EDS) mapping show that the Li‐Naph(s) layer is only ≈ 15 µm thick, and this organic‐based interlayer has an intimate contact with LLZTO without evident gaps (Figure S9, Supporting Information).

Li‐Naph(s) was also proven to have good chemical stability and compatibility with LLZTO. Even stored at elevated temperature (65 °C) for 12 h, Li‐Naph(s) samples remain stable as indicated by the NMR spectra (Figure S11, Supporting Information) and FT‐IR (Figure S12, Supporting Information). Similarly, the mixed sample of LLZTO powder and Li‐Naph(s) (mass ratio = 1: 2) did not show any noticeable changes (Figures S10–S12, Supporting Information). LLZTO has been known for its wide electrochemical stability window (0–6 V).^[^ ^47^, ^48^ ^]^ The electrochemical potential of Li‐Naph(s) is expected to be close to its redox potential in the liquid state (*E* = 0.32 V)^[^ ^49^ ^]^, which explains the high stability of LLZTO against Li‐Naph(s).

**Figure 2:**
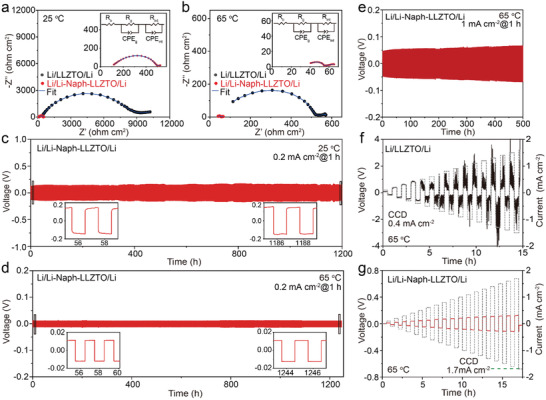
Interfacial resistance measurements of Li/Li‐Naph‐LLZTO/Li cells at a) 25 °C and b) 65 °C, with Li/LLZTO/Li for comparison. Insets are zoom‐in views of Li/Li‐Naph‐LLZTO/Li results. Cycling tests of Li/Li‐Naph‐LLZTO/Li under a current density of 0.2 mA cm^–2^ at c) 25 °C and d) 65 °C. Insets are voltage curves corresponding to different cycling stages. e) Long‐term performance of Li/Li‐Naph‐LLZTO/Li at 65 °C at a high current density of 1 mA cm^–2^. CCD measurements of f) Li/LLZTO/Li and g) Li/Li‐Naph‐LLZTO/Li.

To evaluate the electrochemical performance of this Li‐Naph(s)‐based PMMCI, symmetric Li–Li cells were assembled with or without Li‐Naph(s) interlayer (noted as Li/LLZTO/Li or Li/Li‐Naph‐LLZTO/Li, respectively). The Li‐Naph(s) interlayer was found very effective in improving the interfacial contact between Li metal and the LLZTO solid electrolyte. As indicated by EIS measurements, the interfacial area‐specific resistance (ASR) decreased from 4199.8 to 131.8 Ω cm^2^ at 25 °C (**Figure** **2**a), and from 241.8 to 9.9 Ω cm^2^ at 65 °C (Figure 2b). The ASR values are estimated based on the equivalent circuit in the insets of Figure 2a,b, which shows a good fitting result (Table S2, Supporting Information). *R*_b_, *R*_g_, and *R*_int_ represent the ASRs for garnet bulk, grain boundary, and interface transfer, respectively. CPE_g_ and CPE_int_ denote the constant phase elements paralleled with *R*_g_ and *R*_int_. The corresponding characteristic time constants (*τ*) and capacitance values (*C*) are listed in Table S3 (Supporting Information). *C* and *τ* can be expressed by (R^1−n^CPE)^1/n^ and RC, where *n* is CPE exponent. The capacitance values referring to interfacial transport are in the range of 10^–9^–10^–7^ cm^–2^.^[^ ^31^ ^]^ It's worth noting that the relaxation time is shortened for the interfacial Li^+^ transport process at both 25 and 65 °C for Li‐Naph‐LLZTO, indicating improved electrochemical kinetics. The Li/LLZTO/Li cell shows severe voltage fluctuations and voltage polarization as high as 0.7 V at 0.2 mA cm^–2^ under room temperature (Figure S13, Supporting Information). The quick cell failure is mainly due to the poor contact between Li metal and LLZTO, where ion transport can only occur at limited contact points to induce detrimental Li penetration through the solid electrolyte. In stark contrast, the Li/Li‐Naph‐LLZTO/Li cell exhibits highly stable Li plating/stripping behaviors with a low overpotential of ≈ 148 mV (0.2 mA cm^–2^) at 25 °C for 1200 h (i. e. 600 cycles, Figure 2c). The cell overpotential can be further reduced to ≈12 mV (0.2 mA cm^–2^) at 65 °C and remains high stability for 1200 h, as shown in Figure 2d. The cell impedance also has very limited changes during cycling (Figure S16c, Supporting Information). Besides, the Li/Li‐Naph/LLZTO/Li cell can maintain excellent stability even at a high current density of 1 mA cm^–2^ and capacity of 1 mA h cm^–2^ with a small overpotential of ≈70 mV after 500 h, as demonstrated in Figure 2e. No apparent morphological evolution of the LLZTO surface can be found after disassembling the cycled Li/Li‐Naph‐LLZTO/Li (Figure S17, Supporting Information). In addition, Naph^–^ radical can be observed obviously after cycling at different stages as shown in Figure S18 (Supporting Information), which indicates the retention of a well‐controlled interface. Moreover, we further compared fresh Li‐Naph‐LLZTO as prepared and Li‐Naph‐LLZTO after cycling 100 h. They have almost identical ^1^H NMR spectra (Figure S19, Supporting Information), which proves the excellent stability and compatibility of Li‐Naph with LLZTO.

The excellent electrochemical performance with the Li‐Naph(s)‐based PMMCI lies in the improved effective interfacial contact with the deformable organic interphase, as well as the monolithic nature of this interlayer, which is critical for promoting the uniform Li^+^ flux and electric field at the Li/garnet interface. It is worth mentioning that flat voltage plateaus exhibited in Li symmetric cells at both 25 and 65 °C suggest fast interfacial charge transfer and facial Li nucleation processes with the help of the Li‐Naph(s) interlayer. The fast interfacial charge transfer kinetics can be manifested by the greatly increased CCD with the Li‐Naph(s) interlayer. To get a more fundamental understanding of the effects of PMMCI, we prepared PEO‐LiTFSI (a pure ionic interlayer) and PEO‐Carbon‐LiTFSI (a mixed‐conducting interlayer, weight ratio of carbon is ≈1.4%) coatings on the LLZTO, which noted as PEO‐LLZTO and PEO‐C‐LLZTO, respectively (see preparation routine in Supporting Information). To make a fair comparison, the PEO interlayer thickness was controlled to be 15 µm, same as our PMMCI (Figure S14, Supporting Information). As shown in Figure S15 (Supporting Information), the CCD is slightly increased from 0.4 mA cm^–2^ (bare LLZTO) to 0.5 mA cm^–2^ for the PEO‐LLZTO at 65 °C, and Li/PEO‐C‐LLZTO/Li exhibits a much higher CCD (0.8 mA cm^–2^). This result indicates that the electronic conductivity of PEO‐C‐LLZTO is beneficial for improving interfacial kinetics and suppressing Li dendrite penetration. In addition, the Li/PEO‐C‐LLZTO/Li cell also shows an apparently improved cycling performance at 0.2 mA cm^–2^ under 65 °C, compared to both bare LLZTO and PEO‐LLZTO. These experiment results testify that a uniform interfacial electric field is beneficial for regulating Li^+^ transport and suppressing Li dendrite growth. However, the PEO‐C interlayer still suffers from the low ionic conductivity and intrinsic inhomogeneity, which are unfavorable for uniform Li plating and stripping. In sharp contrast, the CCD reaches up to 1.7 mA cm^–2^ using the Li‐Naph(s) interlayer (Figure 2g; Figure S20, Supporting Information). This plastic and monolithic interlayer with excellent ionic and electronic conducting capabilities enables an intrinsically homogenous electric field and fast Li^+^ transport at the Li/garnet interface, thus significantly reducing the interfacial resistance and achieving uniform and dendrite‐free Li anode plating/stripping. Consistent with the CCD results, the Li‐Naph‐LLZTO cell demonstrates a better rate performance compared to the bare LLZTO that suffered from large polarization and short circuits only after a few hours (Figure S21, Supporting Information). As mentioned before, the Li‐Naph(s) interlayer is stable with both Li anode and LLZTO electrolyte; thus, the improved cell performance is unlikely due to the passivation of the LLZTO by Li‐Naph(s). No apparent effect on the cell stability can be observed after removing the Li‐Naph(s) from the LLZTO pellet (Figure S22, Supporting Information). Additionally, XPS characterizations were conducted to study the influence of Li‐Naph(s) on the composition and structure of SEI on the lithium metal anode before and after cycling, respectively. In the control experiment, the Li/Li‐Naph‐LLZTO/Li cell at open‐circuit voltage without cycling was disassembled to retrieve the Li anode for XPS analysis. As shown in Figure S23 (Supporting Information), the signals of O‐C‐O, lithium alkyl oxides (R–OLi) and –(CH_2_–CH_2_–O)_n_ in the C 1s and O 1s spectra suggest the reactions between Li metal and DME molecules. Upon depth profiling with Ar ions, the ‐(CH_2_–CH_2_–O)_n_ signals disappear. The persistent signals of C═O and Li—O may be due to the pristine surface layer formed on Li metal during the manufacturing process. For the Li anode sample after cycling for 20 h (Figure S24, Supporting Information), the initial XPS spectra show slightly higher signals of C—O and ‐(CH_2_–CH_2_–O)_n_, suggesting slightly increased reactions between Li metal and DME. The –(CH_2_–CH_2_–O)_n_ oligomer may be beneficial for the Li anode by enhancing the elasticity of the PMMCI. However, the XPS signals of the underneath SEI layer upon depth profiling are very close to those of the control sample without cycling, with similar signals of C═O, RO—Li, Li—O, etc. This suggests that the reaction between the Li‐Naph(s) interlayer and Li metal is restricted and explains the stable cell overpotential of Li||Li cell during long‐term cycling (>1000 h, Figure 2d).

**Figure 3:**
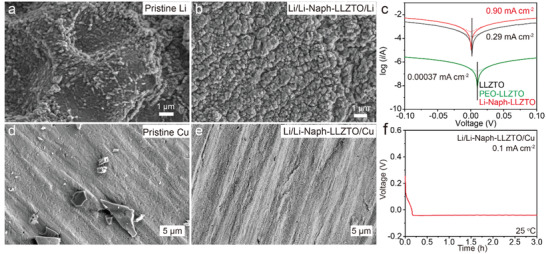
a) Pristine microstructure of Li metal and b) Li/Li‐Naph‐LLZTO/Li after cycling 100 h at 0.2 mA cm^−2^. c) Exchange current densities of Li/LLZTO/Li, Li/PEO‐LLZTO/Li, and Li/Li‐Naph‐LLZTO/Li. SEM images of Cu foils of d) pristine and e) Li‐Naph‐LLZTO after Li deposition. f) Voltage profiles of Li deposition on a Cu substrate at 0.1 mA cm^−2^.

To further illustrate the effect of the homogenized electric field and ion transport at the interface with the PMMCI, the morphologies of lithium deposition on Li anode and copper foil were examined by SEM. The pristine Li exhibited a relatively rough surface with scratches and spines as shown in **Figure** **3**a, while ordered Li metal morphology without Li dendrites was found at the cycled Li surface using the Li‐Naph(s) interlayer (Figure 3b), indicating that the nucleation and growth of Li are well‐controlled and uniform. SEM images of cycled Li electrodes after plating/stripping for 100 h at 0.5 mA cm^–2^ and 1 mA cm^–2^ were also provided, respectively. Li metal at 0.2 mA cm^−2^ shows a relatively dense and smooth morphology, without any dendritic Li (Figure S25, Supporting Information). At high current densities, the overall morphologies of Li deposition were still under control, without any obvious Li dendrite formation (Figure S24b,c, Supporting Information). In addition, the dendrite‐free grain boundary of the LLZTO after cycling 100 h at 1 mA cm^–2^ further confirms the ability of the Li‐Naph(s) to prevent dendrite growth (Figure S25d, Supporting Information). Additionally, the exchange current density at the Li‐Naph(s)/LLZTO interface was estimated to be 0.9 mA cm^–2^, which is threefold higher than the bare LLZTO (0.29 mA cm^–2^) and several orders of magnitude higher than the PEO‐LLZTO (0.00037 mA cm^–2^) in Figure 3c, indicating that the charge transfer kinetics at the interface is greatly improved. More importantly, uniform Li deposition can even be achieved on bare Cu foil with the Li‐Naph(s) interlayer (Figure 3d,e). As shown in Figure S26 (Supporting Information), the cell impedance is significantly reduced compared to that of bare LLZTO. The Li/Li‐Naph‐LLZTO/Cu cell barely exhibits any overpotential in the Li nucleation stage from the voltage curve and a flat dense surface of Li metal can be found on the Cu substrate (Figure 3e,f), which evidences the uniform Li nucleation and growth mode enabled by the Li‐Naph(s) interlayer.

**Figure 4:**
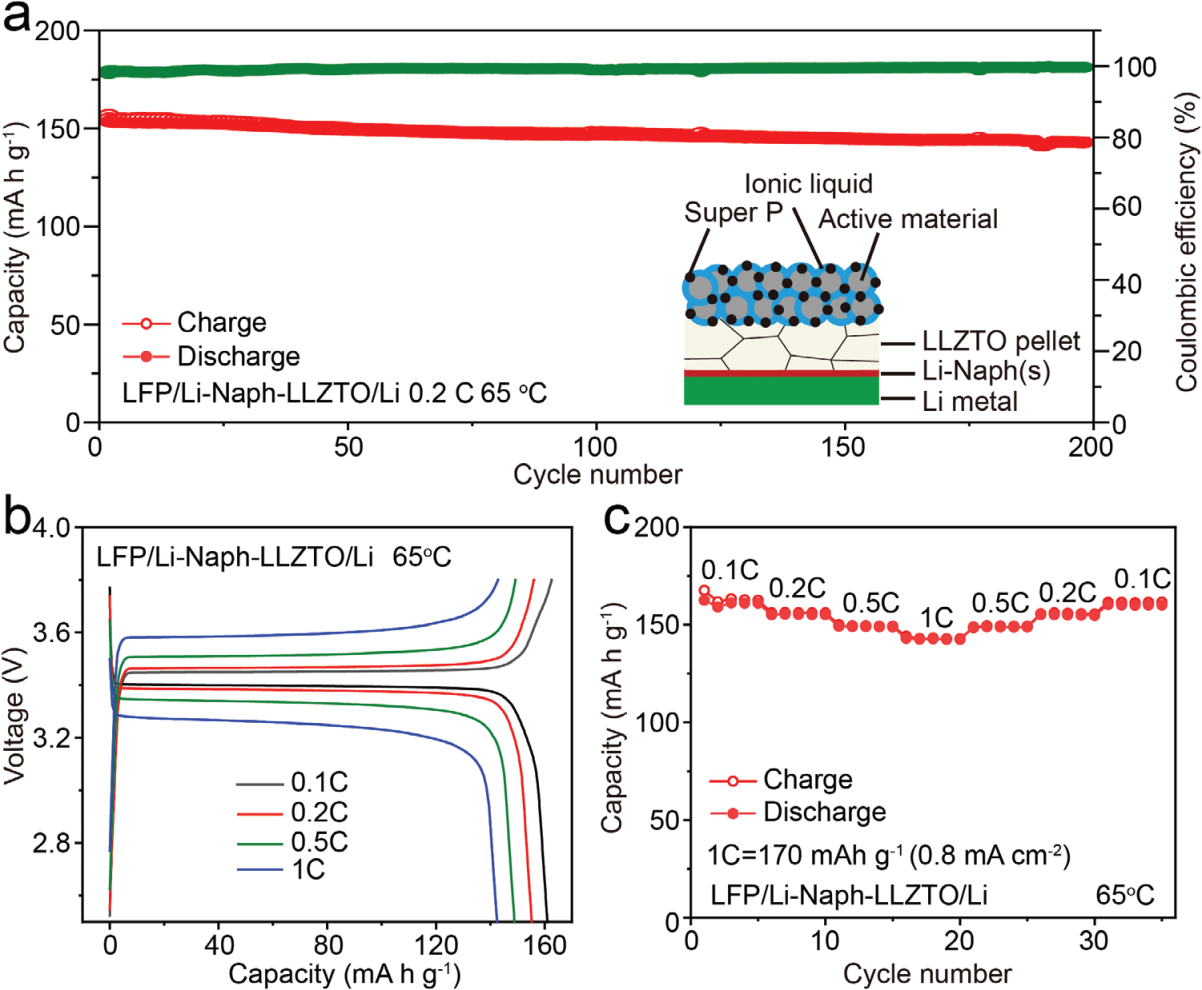
a) Galvanostatic cycling performance of the Li/Li‐Naph‐LLZTO/LFP full cell (Inset: Schematic of the cell). b) Galvanostatic charge and discharge voltage profiles at the current densities from 0.1 C to 1 C and c) corresponding rate performance of the Li/Li‐Naph‐LLZTO/LFP full cell.

The Li‐Naph(s) interlayer has also been integrated into full cells with LiFePO_4_ (LFP) as cathode and Li metal as anode. As shown in **Figure** **4**a, the Li/Li‐Naph‐LLZTO/LFP cell delivers a high capacity retention of 93% after 200 cycles with an average Coulombic efficiency over 99% at 0.2 C (1 C = 170 mA h g^–1^ or 0.8^ ^mA cm^–2^) and 65 °C. Moreover, with the greatly improved charge transfer kinetics at the Li/garnet interface, the full cell also shows a good rate performance with a discharge capacity of 142.5 mA h g^–1^ at 1 C (161.1 mA h g^–1^ at 0.1 C, Figure 4b,c). In sharp contrast, Li/LLZTO/LFP and Li/PEO‐LLZTO/LFP cells exhibit remarkably irreversible capacity, as shown in Figure S27 (Supporting Information).

In addition to its excellent electrochemical performance, the PMMCI also processes unique tunability regarding its electronic state and interfacial charge transfer processes. Other *π*‐conjugating molecules, e.g., biphenyl (Biph) and phenanthrene (Phen), could be used to adjust the energy level of delocalized electrons. It was found that the Li^+^ conductivity is also related to the *π*‐conjugation structure, as shown in Figure S28 (Supporting Information). The Li‐Phen(s) possess the highest Li^+^ conductivity of 7.52 × 10^–3^ S cm^–1^ among the three at RT. The voltage profile of the Li/Li‐Phen‐LLZTO/Li cell is highly smooth and stable with an overpotential < 50 mV (Figure S29, Supporting Information) at 1 mA cm^–2^ and 65 °C, apparently smaller than those found with Li‐Naph and Li‐Biph. Compared to those of Li‐Naph(s) (0.9 mA cm^–2^) and Li‐Biph(s) (0.9 mA cm^–2^), the exchange current density can be further increased three times at the Li‐Phen(s)/LLZTO interface (2.72 mA cm^–2^, Figure S30, Supporting Information). The Li/Li‐Phen(s) interface also shows a much higher exchange current density of 23.6 mA cm^–2^ than those of Li‐Naph(s) (14.22 mA cm^–2^) and Li‐Biph(s) (10.54 mA cm^–2^), as shown in Figure S29d (Supporting Information). The exact reason for variations of electrochemical behaviors for the PMMCI family requires further detailed studies, but is likely to be rooted in the *π*‐conjugation structure, the electronic state, the Li^+^‐coordination environment, the assembly structure, etc. Such versatility of the PMMCI family would offer great opportunities for fabricating tailored interlayers for different solid electrolyte materials.

## 3 Conclusion

In summary, we report a facile interface engineering approach for solid‐state batteries with PMMCI using solid‐state lithium naphthalenide. The Li‐Naph(s) modified LLZTO exhibits a high CCD of 1.7 mA cm^–2^, small overpotentials (as low as 12 mV), excellent long‐term cycle stability over 1200 h under room, and elevated temperatures at 0.2 mA cm^–2^. The PMMCI also enables a superior rate capability and maintains stable cycling for 500 h at a high current density of 1 mA cm^–2^. The significant improvement in the electrochemical performance is mainly attributed to the excellent interfacial Li^+^ and e^–^ charge transfer properties of the PMMCI. The PMMCI not only allows the close contact between Li anode and the garnet electrolyte, but also provides ordered layered frameworks for facial transportations of both Li^+^ (*σ*_Li_^+^ = 4.38 × 10^–3^ S cm^–1^) and electrons (*σ*_e‐_ = 1.01 × 10^–3^ S cm^–1^). Because of the homogenized Li^+^ fluxes and electric field at the Li/garnet interface, this monolithic interlayer greatly improves the interfacial charge transfer kinetics and empowers dendrite‐free Li plating/stripping at high rates. This PMMCI design highlights the importance of controlling the ionic and electronic properties simultaneously for addressing the solid–solid interfacial challenges and opens new opportunities for the development of practical solid‐state batteries (including Na–K metal anodes).

## 4 Experimental Section

### Preparation of LLZTO Pellets

The Li_6.4_La_3_Zr_1.4_Ta_1.6_O_12_ (LLZTO, Hefei Kejing, China) garnet powder was pressed into pellets of 13 mm diameter for calcination at 1170 °C for 6 h covered with the same powder. To remove Li_2_CO_3_ and LiOH residues from the surface of pellets introduced during the preparation process, LLZTO pellets were first polished by 400‐grit sandpaper. Then, the pellets were immersed into 1 m HCl for 30 s, rapidly washed by ethanol, and dried by a hairdryer to almost eliminate the impurities. The LLZTO pellets as prepared were kept in the Ar‐filled glovebox for further use.

### Preparation of Li‐Naph, Li‐Phen, and Li‐Biph

Li‐Naph solution(1 m) was obtained as literature reported. Typically, 10 mmol naphthalene (Naph, ≥98%, Aladdin) was first dissolved into 1,2‐dimethoxyethane (DME), and Li foils (10 mmol) were added into the above solution and rested over 1 h to make it dissolve completely. And the obtained dark green liquid, which is noted as Li‐Naph (l). Li‐Phen and Li‐Biph were obtained in the same way. For further characterization, Li‐Naph, Li‐Phen, and Li‐Biph were vacuum dried in the antechamber of the glovebox for 5 and 15 min at room temperature to obtain corresponding solids.

### Preparation of Li‐Naph‐LLZTO Pellets

Ten microlitres of Li‐Naph (l) was carefully dropped on the LLZTO pellets, followed by vacuum drying for 15 min to get Li‐Naph‐LLZTO pellets. Correspondingly, Li‐Phen‐LLZTO and Li‐Biph‐LLZTO pellets were fabricated by a similar method. For better comparison, LLZTO powders and Li‐Naph(s) powders with a mass ratio of 1:2 were mixed and grounded homogeneously for further characterizations.

### Preparation of PEO‐LLZTO Pellets

LiTFSI (0.407 g) and 1 g PEO (nLi:nEO = 1:16) were dissolved in 15 mL anhydrous acetonitrile and stirred at 70 °C for 12 h. Then, 10 µL PEO solution was carefully dropped on the LLZTO pellets and dried by a hairdryer to remove almost all the solvent, followed by drying at 70 °C for 12 h in a vacuum oven to get PEO‐LLZTO pellet.

### Preparation of PEO‐C‐LLZTO Pellets

Super P (20 mg) was added into the PEO solution, followed by the same procedure described above to get the PEO‐C‐LLZTO pellet (weight ratio of C is ≈1.4%).

### Electrochemical Measurements

LLZTO pellets were coated by Ag paste, followed by a solidification process at 150 °C under vacuum for 3 h to remove organic components and ensure tight contact. Then, Ag/LLZTO/Ag blocking cell was measured by electrochemical impedance spectroscopy (EIS) to calculate the ionic conductivity of LLZTO pellets and activated energy (*E*_a_) in a frequency range from 1 Hz to 20 MHz with an AC amplitude of 10 mV on a Solartron frequency analyzer. Exchange current density was obtained based on the following cells structures: Li/Li‐Naph/Li, Li/Li‐Phen/Li, Li/Li‐Biph/Li, Li/Li‐Naph‐LLZTO/Li, Li/Li‐Phen‐LLZTO/Li, Li/Li‐Biph‐LLZTO/Li, Li/LLZTO/Li, Li/ZnO‐LLZTO/Li, and Li/PEO/Li.

### Lithium Symmetric Cell Assembly

2032 coin‐type cells were assembled to evaluate electrochemical performance. Typically, Li foils (≈10 mm in diameter) were placed on both sides of the LLZTO pellet, and stainless steel spacer was put on one side of Li to ensure good contact. The EIS spectra of symmetric cells were also measured to estimate the interface area‐specific resistance (ASR) with an applied frequency range from 100 mHz to 1 MHz at RT or 65 °C on a VMP3 potentiostat/galvanostat station (Bio‐logic Science Instruments). Cycling tests were performed at a current density of 0.2 mA cm^–2^ or 1 mA cm^–2^ with fixed plating/stripping time of 1 h. For rate performance measurement, these cells were performed under the current densities ranging from 0.05 to 1.2 mA cm^−2^ with a fixed plating/stripping interval of 0.5 h. CCD measurement was employed under the stepped current density from 0.1 mA cm^−2^ (1 h per cycle, 0.2 mA cm^−2^ per step). The cells were discharged–charged under the galvanostatic condition on a Land battery test system (Wuhan, China).

### Lithium Asymmetric Cell Assembly

The asymmetric cell was constructed using a Cu electrode disc (diameter ≈12 mm), a Li metal chip (250 µm thickness with diameter ≈10 mm) as the anode. Li foil was placed on one side of the LLZTO pellet, and Cu foil was put on the other side. Then, a flexible Ni foam was placed on the Cu foil and a steel spacer was put on the Li foil to ensure intimate contact. The asymmetric cell was discharged at a current density of 0.1 mA cm^−2^ and deposited for 3 h.

### Full Cell Assembly Based on LiFePO_4_

The LiFePO_4_ (LFP) cathode was prepared as the following procedure: The LFP was mixed with super P carbon black and PVDF binder in *N*‐methyl‐1,2‐pyrrolidinone (99%, Sigma–Aldrich) with a mass ratio of 80:10:10 to form a slurry. The slurry was coated onto Al foil current collectors and dried at 80 °C for 12 h under vacuum. The mass loading of the active materials is 4 mg cm^–2^. Then a Li‐Naph‐LLZTO pellet (with Li‐Naph(s) only on the anode side) was placed on top of the LFP cathode, followed by a piece of lithium‐metal chip and nickel foam in sequence. To improve the contact with Li metal and LFP cathode, 5 µL ionic liquid (PY14TFSI, Sigma–Aldrich) was added to the cathode. The cells were charged–discharged under the galvanostatic condition on a battery test system between 3.8 and 2.4 V at 65 °C under 0.2 C (1 C = 170 mA h g^–1^).

### Structure Characterization

EPR spectra were recorded on a JES‐FA200 (JEOL) spectrometer. The liquid sample was taken out by a capillary (borosilicate glass, 0.8–1.1 × 100 mm) and the concentration of Li‐Naph liquid was 10^–2^ m. Solid samples were taken out by conventional nuclear tubes and then recorded by EPR spectrometer at indicated temperature and parameters. Correction of g‐factor obeyed the following equation: *g* = 0.07145 ×  *γ* (MHz)/*H* (mT), where *γ* refers to microwave frequency, and *H* is resonance field. PXRD characterization was performed on an X`Pert MPD Philips X‐ray diffraction meter with filtered Cu K*α* radiation (*λ* = 1.5405 Å), and all air‐sensitive samples were performed by covering a Kapton film. Scanning electron microscopy images were obtained on a GeminiSEM 500 operating at 3 kV.

## Conflict of Interest

The authors declare no conflict of interest.

## Supporting information

Supporting information

## Data Availability

The data that support the findings of this study are available from the corresponding author upon reasonable request.
