# Supplementary material for: Plastic Monolithic Mixed‐Conducting Interlayer for Dendrite‐Free Solid‐State Batteries
Source: Adv Sci (Weinh). 2022 Apr 28;9(18):2105924. doi: 10.1002/advs.202105924 (PMC9218657; doi:10.1002/advs.202105924)
Supplement: Supplementary file 1 — Supporting information [file ADVS-9-2105924-s001.pdf]

ADVANCED SCIENCE
Open Access

Supporting Information

for *Adv. Sci.*, DOI 10.1002/advs.202105924

Plastic Monolithic Mixed-Conducting Interlayer for Dendrite-Free Solid-State Batteries

*Bing-Qing Xiong, Shunqiang Chen, Xuan Luo, Qingshun Nian, Xiaowen Zhan, Chengwei Wang and Xiaodi Ren**

Supporting Information

Plastic Monolithic Mixed-Conducting Interlayer for Dendrite-Free Solid-State Batteries

*Bing-Qing Xiong, Shunqiang Chen, Xuan Luo, Qingshun Nian, Xiaowen Zhan, Chengwei Wang, Xiaodi Ren**

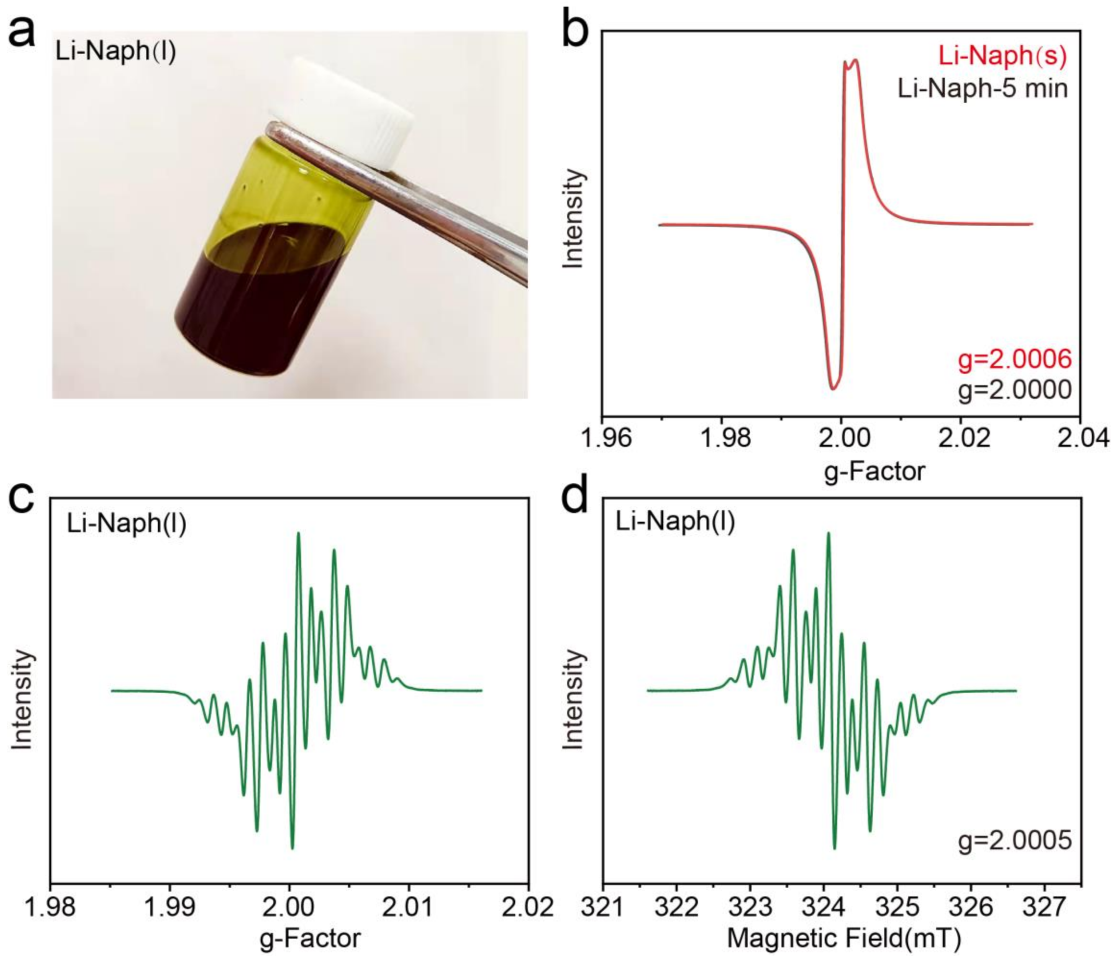

Figure S1. (a) Photographs of the Li-Naph solution, EPR spectra of (b) Li-Naph vacuumed for 5 min and 15 min (Li-Naph(s)). EPR spectra of (c-d) lithium naphthalenide solution.

Note: After a vacuum-drying process at room temperature for 5 min, free/volatile DME was removed, and EPR characteristics of solid-state Li-Naph appeared. After vacuum drying for 15 min, the EPR signal remains unchanged, which indicates that the absence of free DME solvent in the crystalline Li-Naph(s) and the remaining DME molecules are coordinated with Li^+.

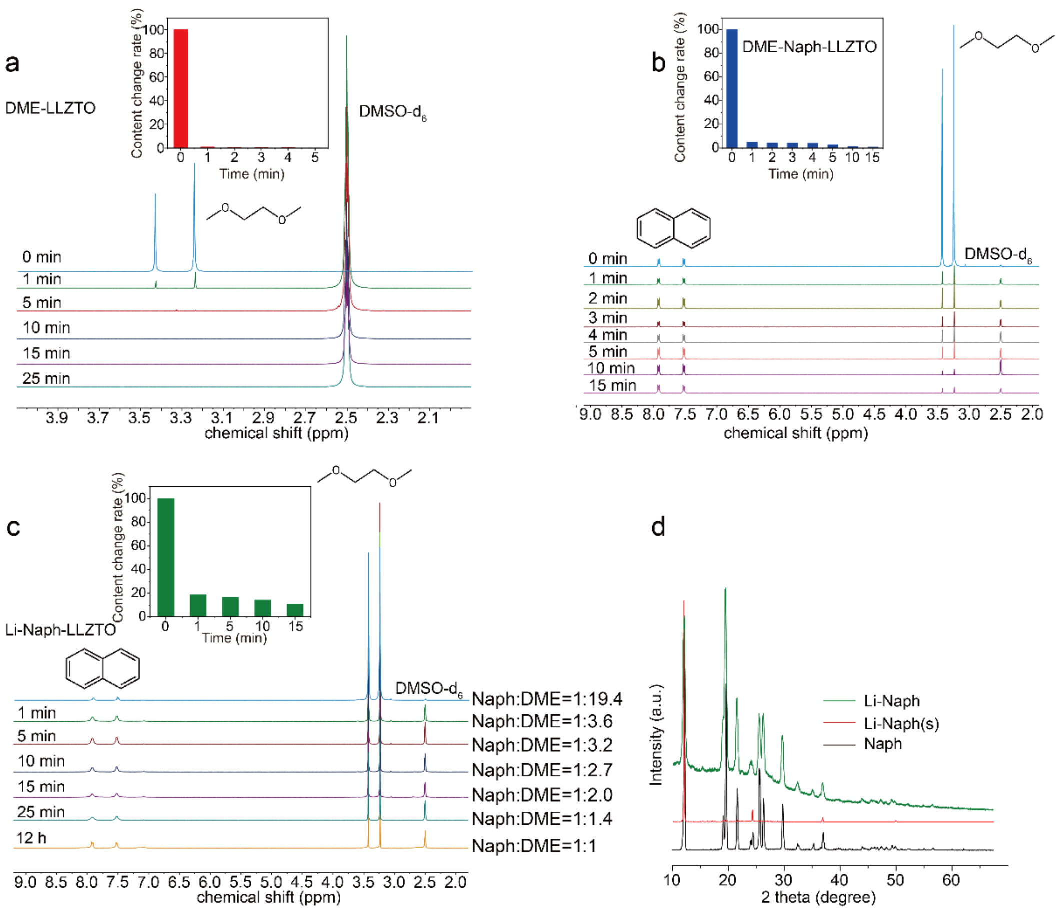

Figure S2. ^{1}H NMR spectra of (a) DME-LLZTO, (b) DME-Naph-LLZTO and (c) Li-Naph-LLZTO vacuumed for a different time before dissolved in DMSO-d_6 (Inset: Content changes of the DME molecules in vacuuming for a different time. We defined the content of the DME molecule as 100% for 0 min.). (d) XRD patterns of Naph, Li-Naph(s) (Naph: DME=1:2, molar ratio), Li-Naph (Naph: DME=1:4).

Note: To make a better comparison, we dropped DME on the LLZTO (noted as DME-LLZTO) with the same amount as Li-Naph-LLZTO and then vacuum-dried the samples for 1 min, 2 min, 3 min, 4min, 5 min, 10 min, and 15 min, respectively. The samples were then rinsed in DMSO-d_6 for NMR analysis. As shown in Figure S2a, the ^{1}H-NMR signals of DME molecules decreased significantly (Figure S2a inset) within 1 min under vacuum and almost completely vanished after 5 min due to the volatile nature of DME (boiling point of 85°C). We also compare the evaporation rate of DME with the presence of naphthalene (without the addition of Li). As shown in Figure S2b, there is barely any DME signals observed after vacuum-drying after 15 min. As a result, free DME solvent can be almost fully eliminated within the first few minutes of a vacuum-drying process during the Li-Naph(s) preparation process. Further removing the coordinated DME molecules in the crystalline structure requires considerable longer time

duration under vacuum. As shown in Figure S2c, the ratio between Naph and DME remains 1:1 after 12 h under dynamic vacuum.

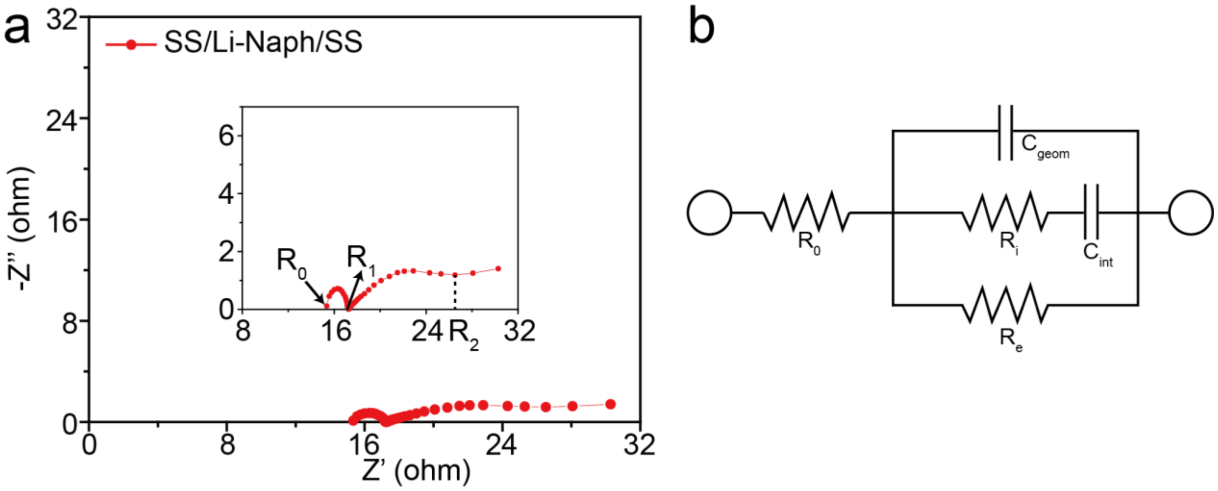

Figure S3. (a) EIS measurement of SS/Li-Naph/SS (1 MHz~10 μHz) and (b) corresponding equivalent circuit.

To separate the electronic and ionic resistance, the Li-Naph solution was cautiously dropped on the stainless steel disc and vacuumed for 15 min to assemble a symmetric SS/Li-Naph/SS cell. For SS/Li-Naph/SS, the thickness of Li-Naph(s) layer is 0.2 mm, and its diameter is 15 mm. The ionic conductivity is from σ_i=L/S×R_i, and electric conductivity is from σ_e=L/S×R_e, where S and L refer to surface area and thickness of the Li-Naph(s) layer.

According to Huggins's EIS method, mixed conducting materials exhibited two semicircles in the complex plane. In the equivalent circuit (Figure S2b), R_0 represents the contact resistance, R_i represents ionic resistance, and R_e refers to electronic resistance, C_{geom} is geometric capacitance. At lower frequencies, the equivalent circuit should be interpreted as a parallel combination of electronic resistance with geometric capacitance. At higher frequencies, the impedance due to the relatively large C_{int} becomes insignificant and is not observed in the measurements. As a result, the equivalent circuit at higher frequencies is a parallel arrangement with three legs: geometric capacitance C_{geom} with two resistances (R_i and R_e in parallel). There are two intercepts on the horizontal axis of the impedance spectrum (Figure S2a), noted as R_1 and R_2. (R_2-R_0) refers to R_e. and (R_1-R_0) results from the parallel combination of R_e and R_i, which meets the following equation: 1/(R_1-R_0) = l/R_i + 1/R_e. Therefore, both electronic and ionic conductivities can be estimated by the Huggins method.

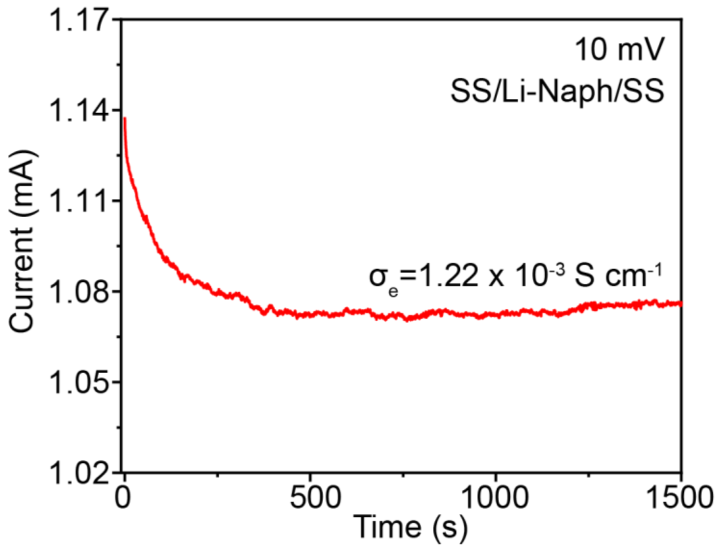

Figure S4. The Current-time curve of SS/Li-Naph/SS cell under DC polarization at 10 mV.

To validate the conductivity results from the Huggins method, DC polarization was also carried out to test the electronic conductivity of the Li-Naph(s). As shown in Figure S4, under a DC polarization of 10 mV, the current stabilizes at ~ 1.075 mA. After the long-term DC polarization, the current should be only from electronic conduction with no net flow of Li^+ ions. The electronic conductivity of the Li-Naph(s) is calculated to be 1.22×10^{-3} S cm^{-1}, which is close to the value of 1.01×10^{-3} S cm^{-1} obtained from the Huggins method.

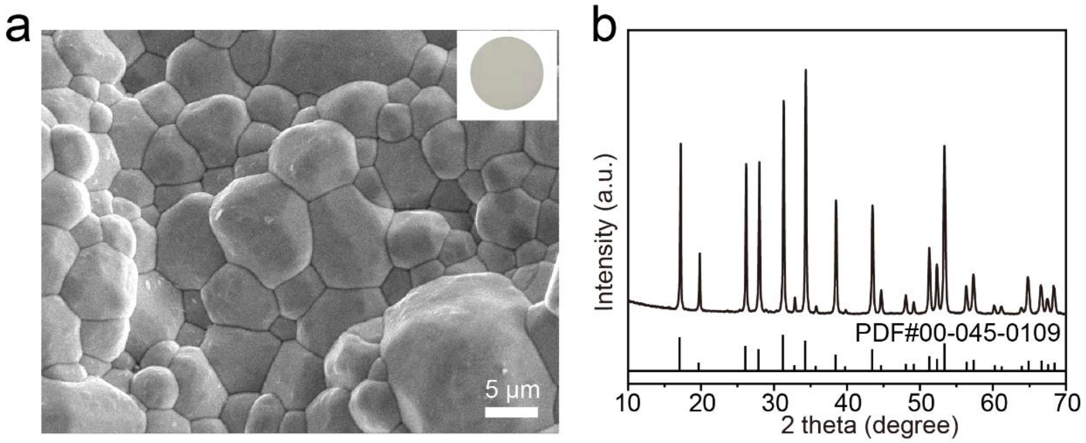

Figure S5. (a) Cross-sectional SEM image and (b) PXRD patterns of LLZTO pellet. Inset is the digital photograph of LLZTO pellet.

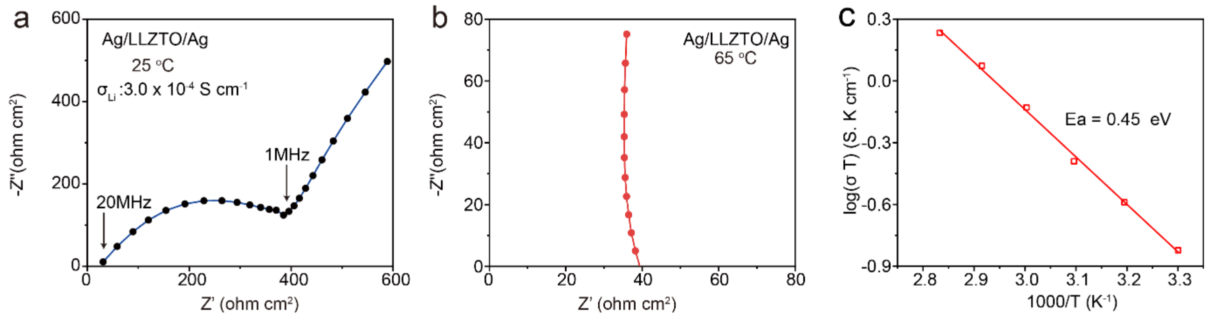

Figure S6. EIS spectrum of the LLZTO pellet at (a) 25 ^{o}C and (b) 65 ^{o}C. (c) Arrhenius plot of the ionic conductivity of LLZTO.

Note: Garnet ASR was obtained from the EIS garnet conductivity measurement of the Ag/LLZTO/Ag symmetric cells, and total garnet ASR is 480.3 Ω cm^2 at 25 ^{o}C and 39.3 Ω cm^2 at 65 ^{o}C.

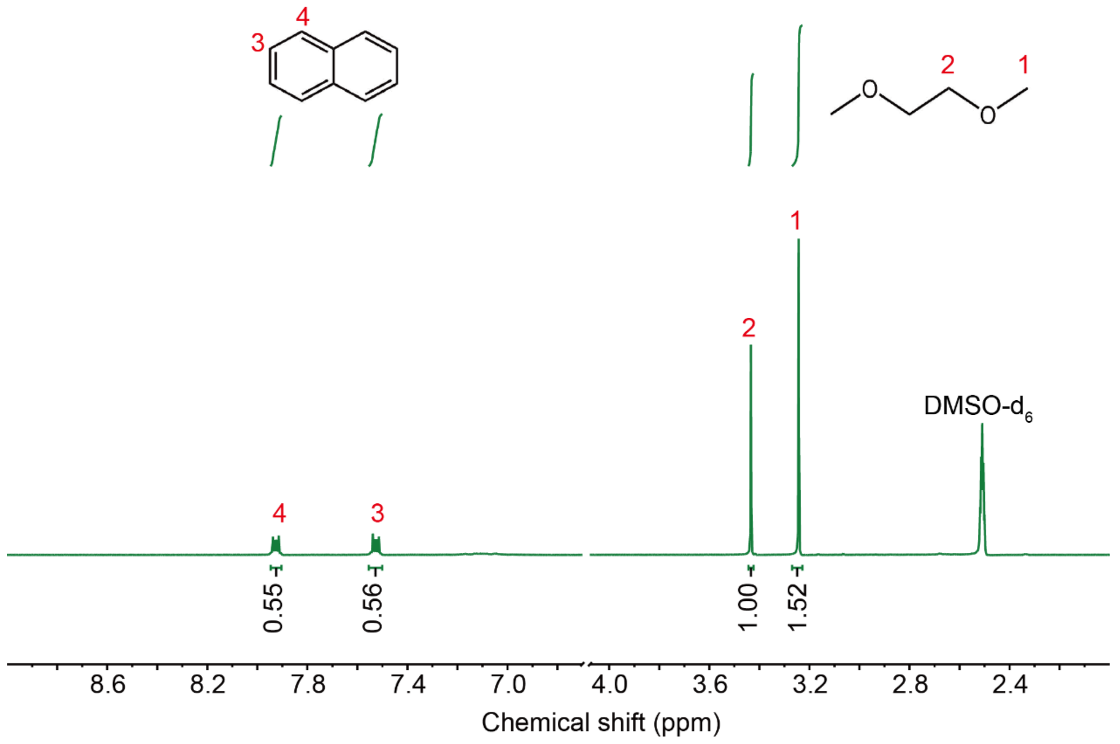

Figure S7. ^{1}H NMR spectrum of Li-Naph (s) dissolved in DMSO-d_6.

Note: From the integral area of the peak, it can be calculated that the mole ratio of Naph and DME in the Li-Naph(s) is 1:2.

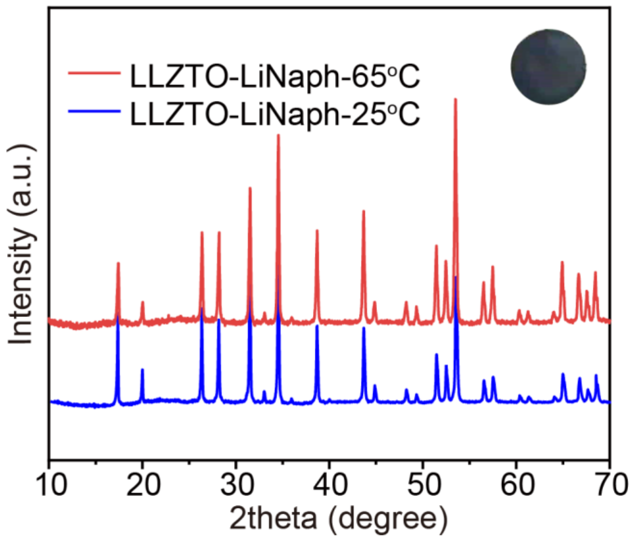

Figure S8. PXRD patterns of Li-Naph(s) modified LLZTO pellet at 25 ^{o}C and 65 ^{o}C.

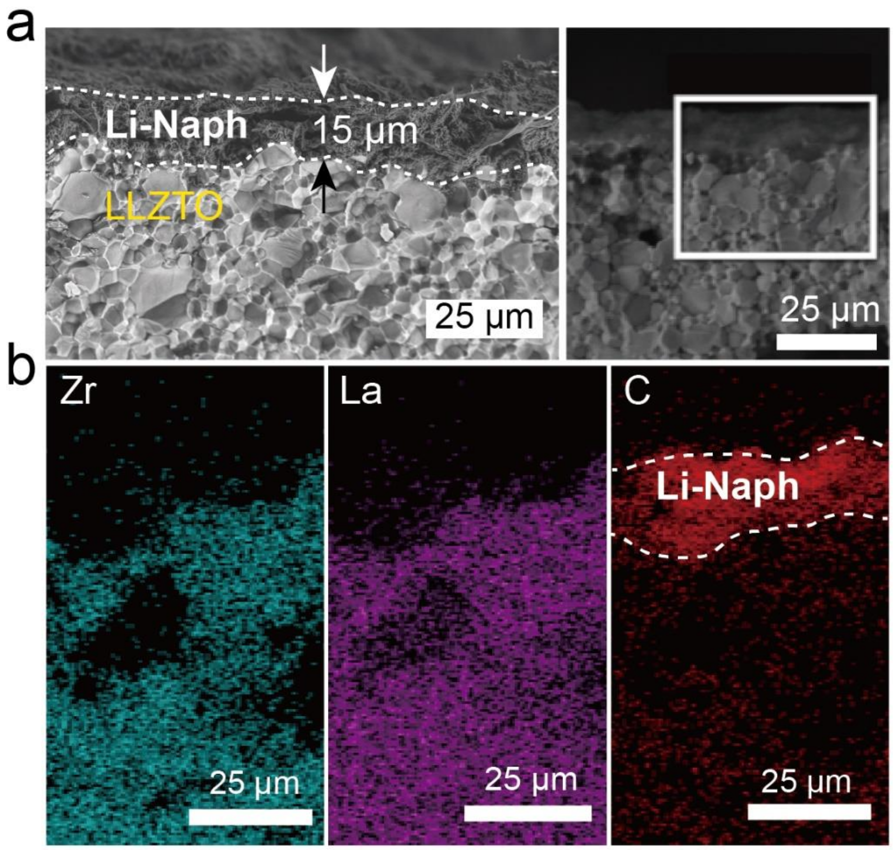

Figure S9. (a-b) cross-sectional SEM images and corresponding EDS mapping of Li-Naph-LLZTO.

Note: To prepare the cross-section sample, Li/Li-Naph-LLZTO/Li was first assembled under a pressure of 50 MPa to break the Li-Naph-modified LLZTO pellet. Then the cell was disassembled, and a fragment of Li-Naph-LLZTO was chosen to do cross-sectional SEM characterization.

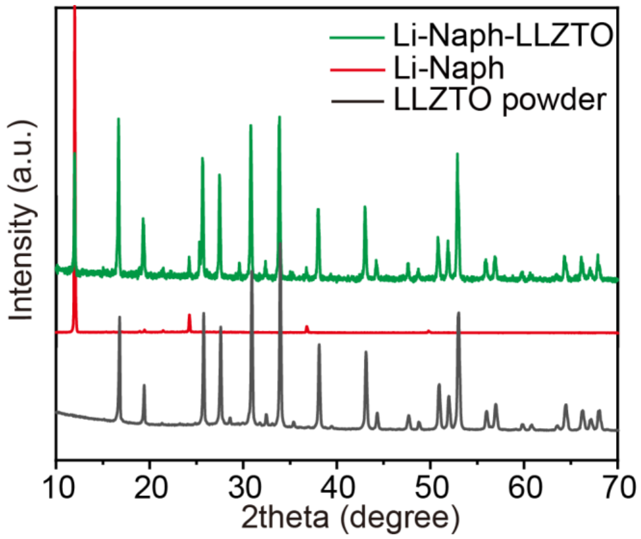

Figure S10. PXRD patterns of LLZTO and Li-Naph mixed powders.

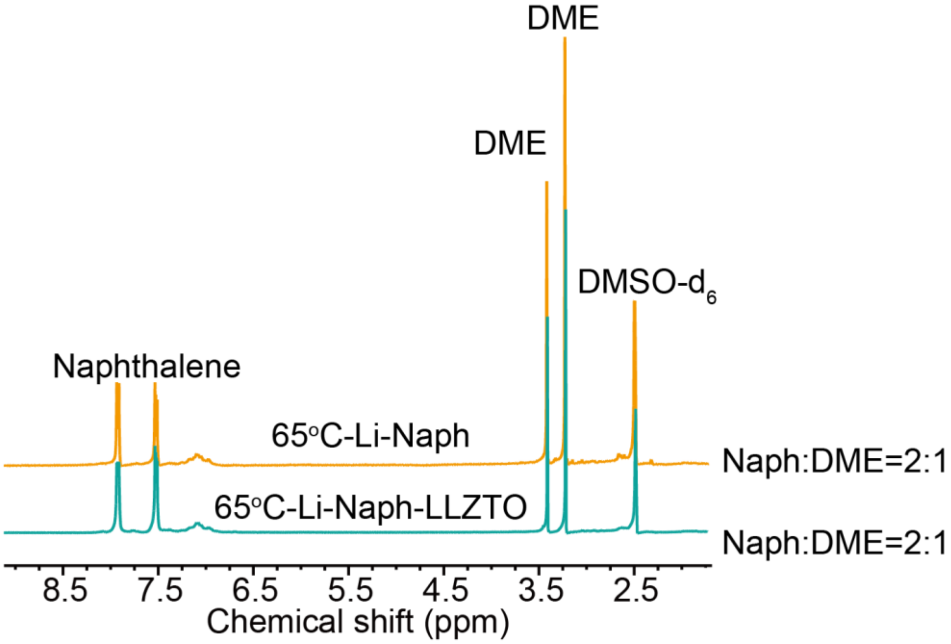

Figure S11. ^{1}H NMR spectrum of Li-Naph (s) in DMSO-d_6 after heated for 12 h at 65 ^{o}C with or without LLZTO powder.

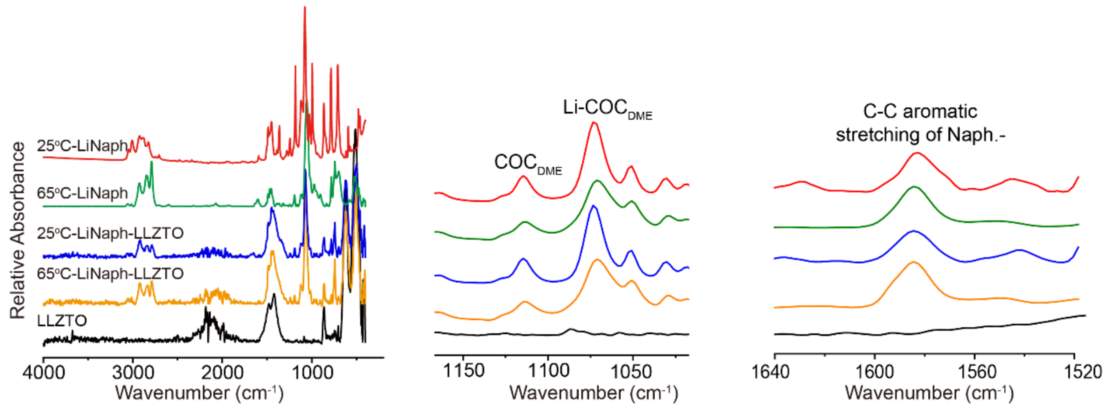

Figure S12. FT-IR spectra of Li-Naph and Li-Naph-LLZTO powders heated for 12 h at 25 °C and 65 °C, respectively.

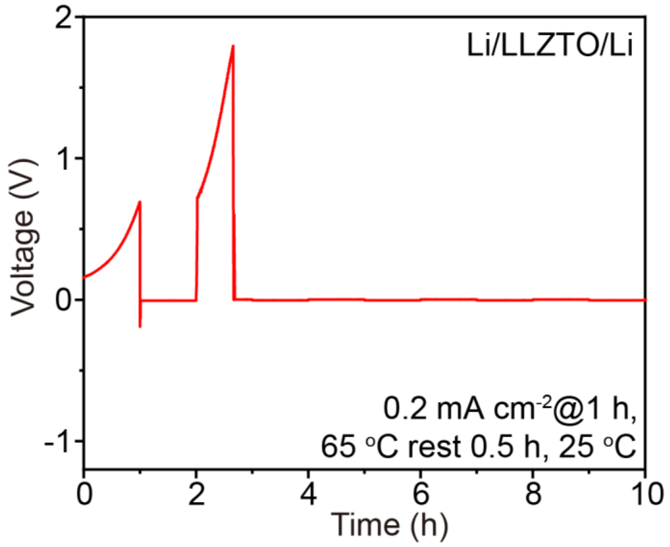

Figure S13. Li/LLZTO/Li cell at a current density of 0.2 mA cm^{-2} with plating/stripping time of 1 h (0.2 mAh cm^{-2}) at 25 °C.

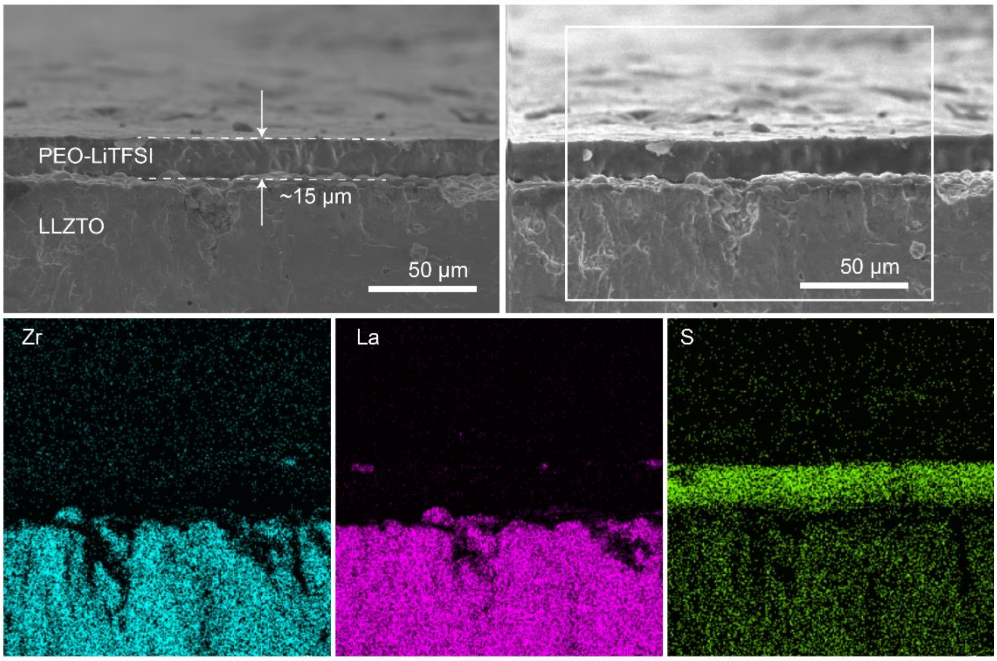

Figure S14. (a-b) cross-sectional SEM images and corresponding EDS mapping of Li-PEO-LLZTO.

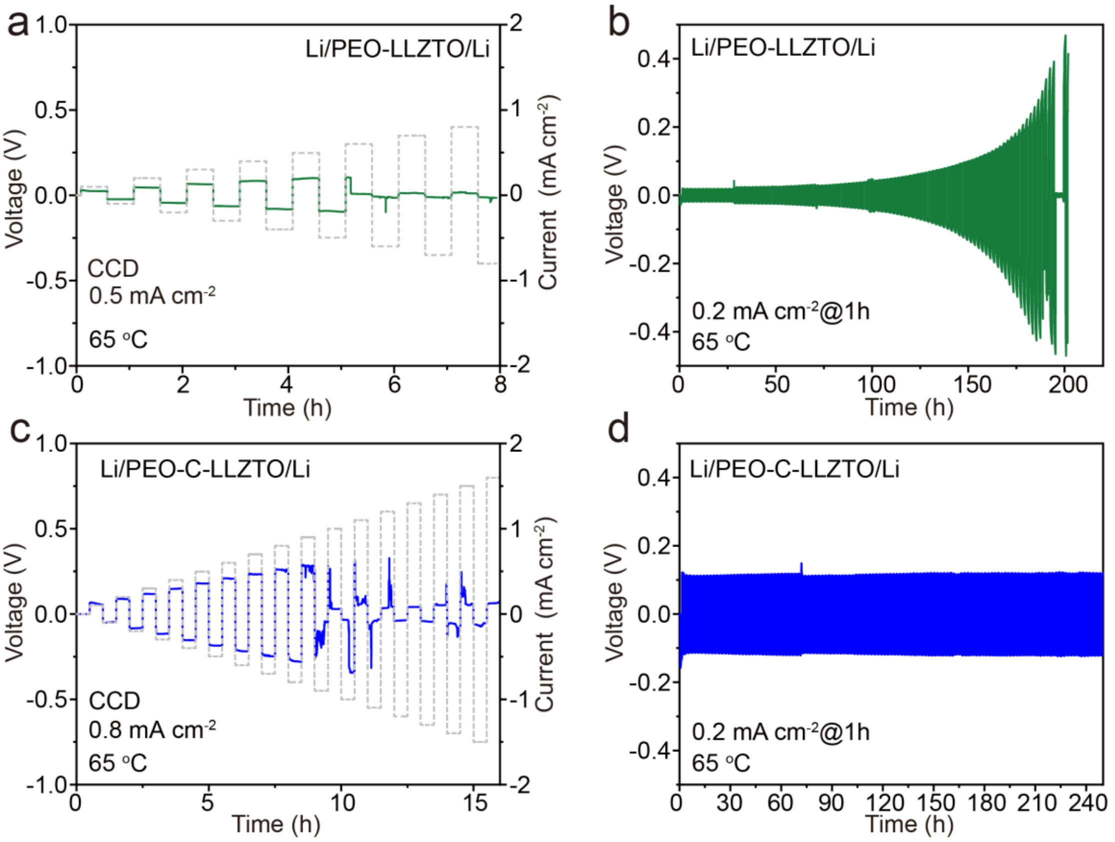

Figure S15. CCD measurements (a) Li/PEO-LLZTO/Li and (c) Li/PEO-C-LLZTO/Li; long-term performance of (b) Li/PEO-LLZTO/Li and (d) Li/PEO-C-LLZTO/Li at 65 ^{o}C under a current density of 0.2 mA cm^{-2}.

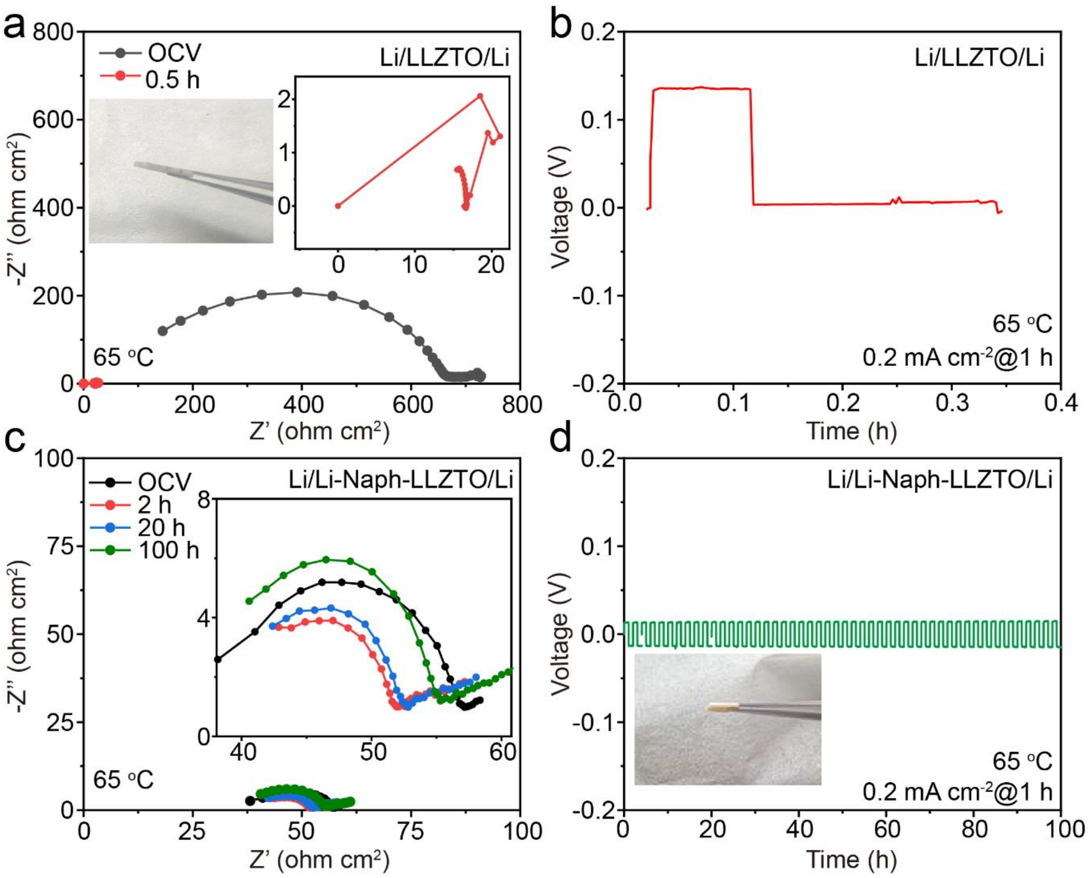

Figure S16. EIS measurements of (a) Li/ LLZTO/Li (inset: photo of LLZTO after Li penetration) and (c) Li/Li-Naph-LLZTO/Li symmetric cells. Galvanostatic cycling of (b) a symmetric Li/LLZTO/Li and (d) Li/Li-Naph-LLZTO/Li cell at a current density of 0.2 mA cm^{-2} with plating/stripping time of 1 h (0.2 mAh cm^{-2}) (inset: photo of Li-Naph-LLZTO after cycling 100 h).

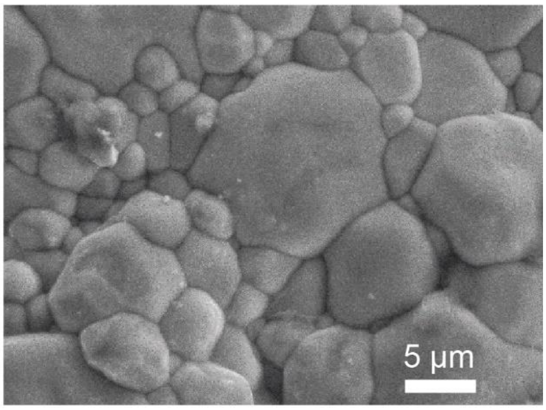

Figure S17. Surface morphology of Li-Naph-LLZTO pellets after cycling 100 h at 0.2 mA cm^{-2} (0.2 mAh cm^{-2}).

Note: For SEM measurement on Li-Naph-LLZTO after cycling 100 h, the Li/Li-Naph-LLZTO/Li cell was dissembled. Both the LLZTO pellet and Li were cautiously taken and immersed into DME to remove Li-Naph(s) on the surface. Then, the LLZTO and Li were vacuum dried for further characterizations.

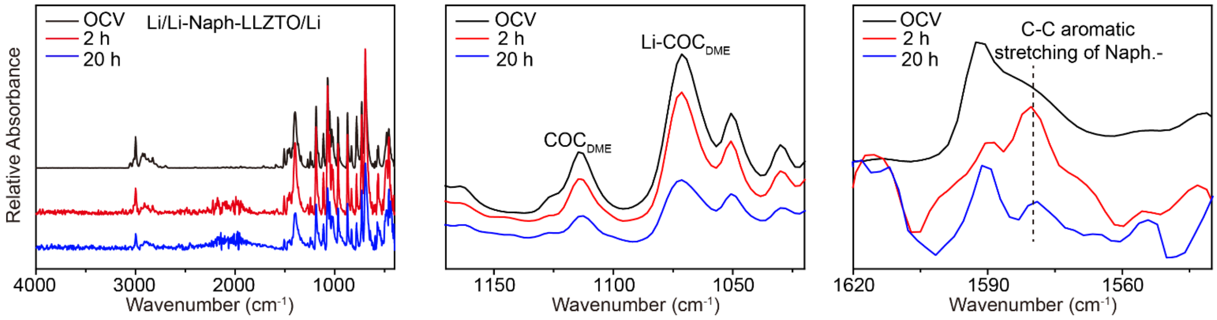

Figure S18. FT-IR spectra of Li foils at different stages in Li/Li-Naph-LLZTO/Li symmetric cells.

Note: For FT-IR measurement on Li-Naph-LLZTO at different stages, the Li/Li-Naph-LLZTO/Li cell was dissembled, and Li foils were directly characterized.

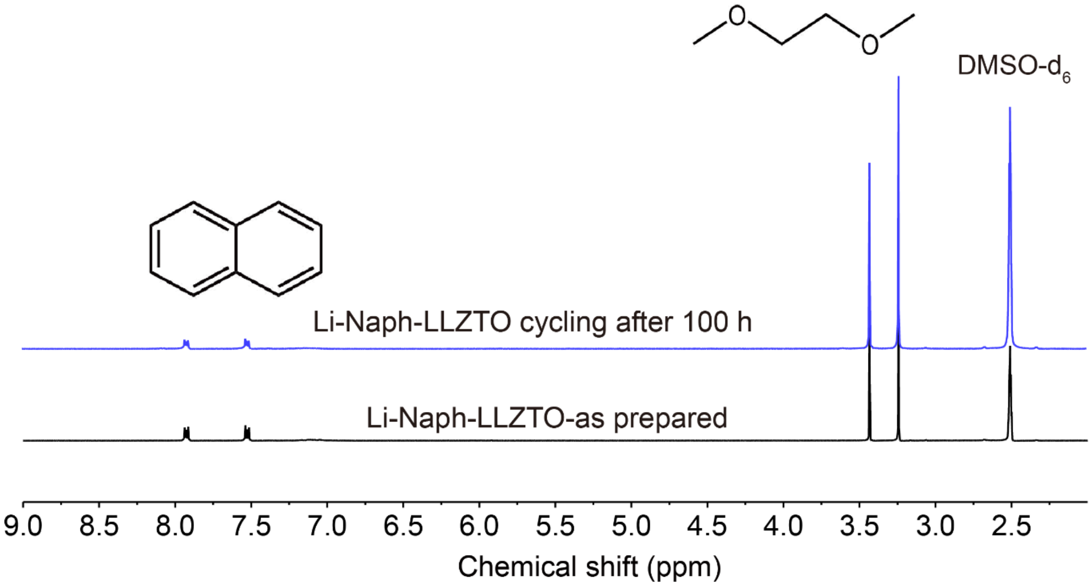

Figure S19. ^{1}H NMR spectra of Li-Naph-LLZTO as prepared and cycling after 100 h.

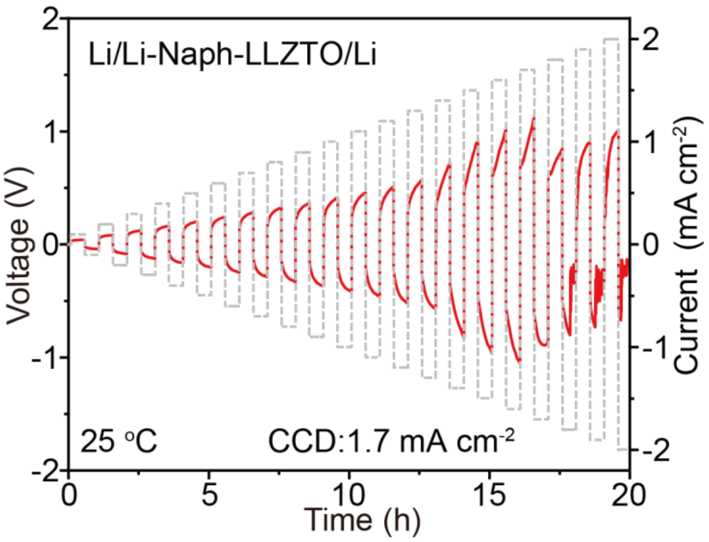

Figure S20. CCD measurement of Li/Li-Naph-LLZTO/Li at 25 °C.

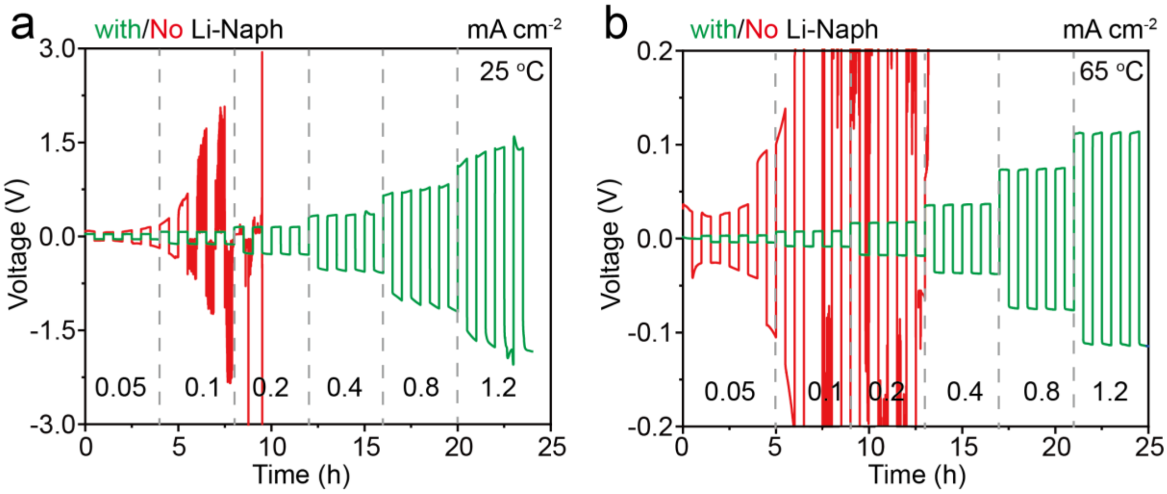

Figure S21. Comparison of rate performance of Li/LLZTO/Li and Li/Li-Naph-LLZTO/Li symmetric cells from 0.05 to 1.2 mA cm^{-2} at (a) 25 ^oC and (b) 65 ^oC.

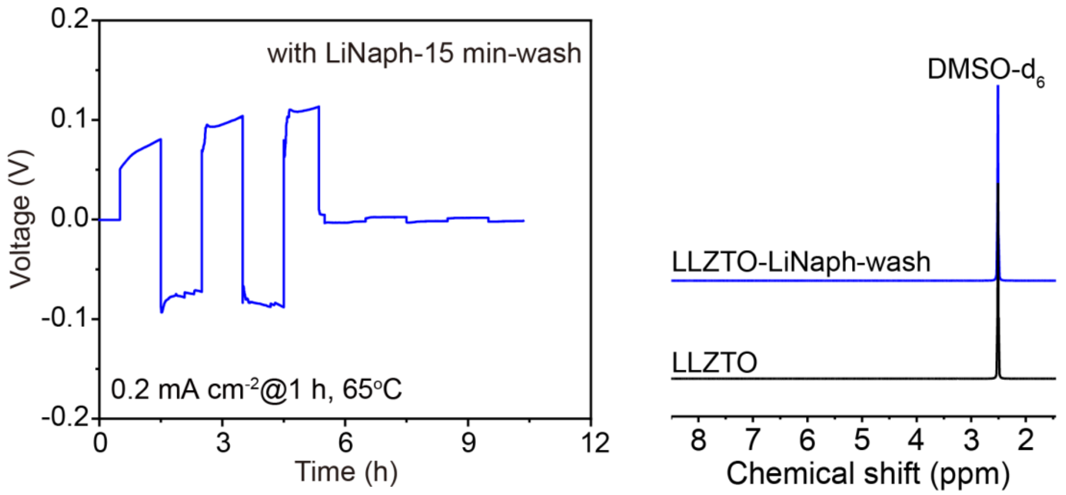

Figure S22. Li/Li-Naph-wash-LLZTO/Li cell at a current density of 0.2 mA cm^{-2} with plating/stripping time of 1 h (0.2 mAh cm^{-2}) at 25 ^oC and ^1H NMR spectrum of Li-Naph-LLZTO-wash and LLZTO in DMSO-d_6, respectively.

Note: To understand more about the role of Li-Naph, Li-Naph on the Li-Naph-LLZTO was firstly eliminated (noted as Li-Naph-LLZTO-wash) to distinguish whether the performance improvement is due to possible new interphase formed between Li-Naph and LLZTO. ^1H-NMR verified that Li-Naph was completely removed with the signal of Naph vanished (Figure S15b). The voltage profile of Li/Li-Naph-LLZTO-wash/Li under a current density of 0.2 mA cm^{-2} at 65 ^oC was given, which shows a smaller overpotential compared with bare LLZTO (Figure S11) but limited cycling life (Figure S15a). This phenomenon demonstrates that the possible new interphase has no obvious effect on the Li anode stability and the excellent performance in Fig 2 is mainly attributed to the uniform Li^+ and e^- fluxes provided by the Li-Naph(s) interlayer.

Prepared of Li-Naph-LLZTO-wash:

Li-Naph-LLZTO was washed by DME over 3 times until the dark-green color vanished, followed by vacuum drying at glove-chamber for 12 h, which is noted as Li-Naph-LLZTO-wash. Subsequently, Li-Naph-LLZTO-wash was assembled with Li anode to form Li symmetric cell for galvanostatic measurement.

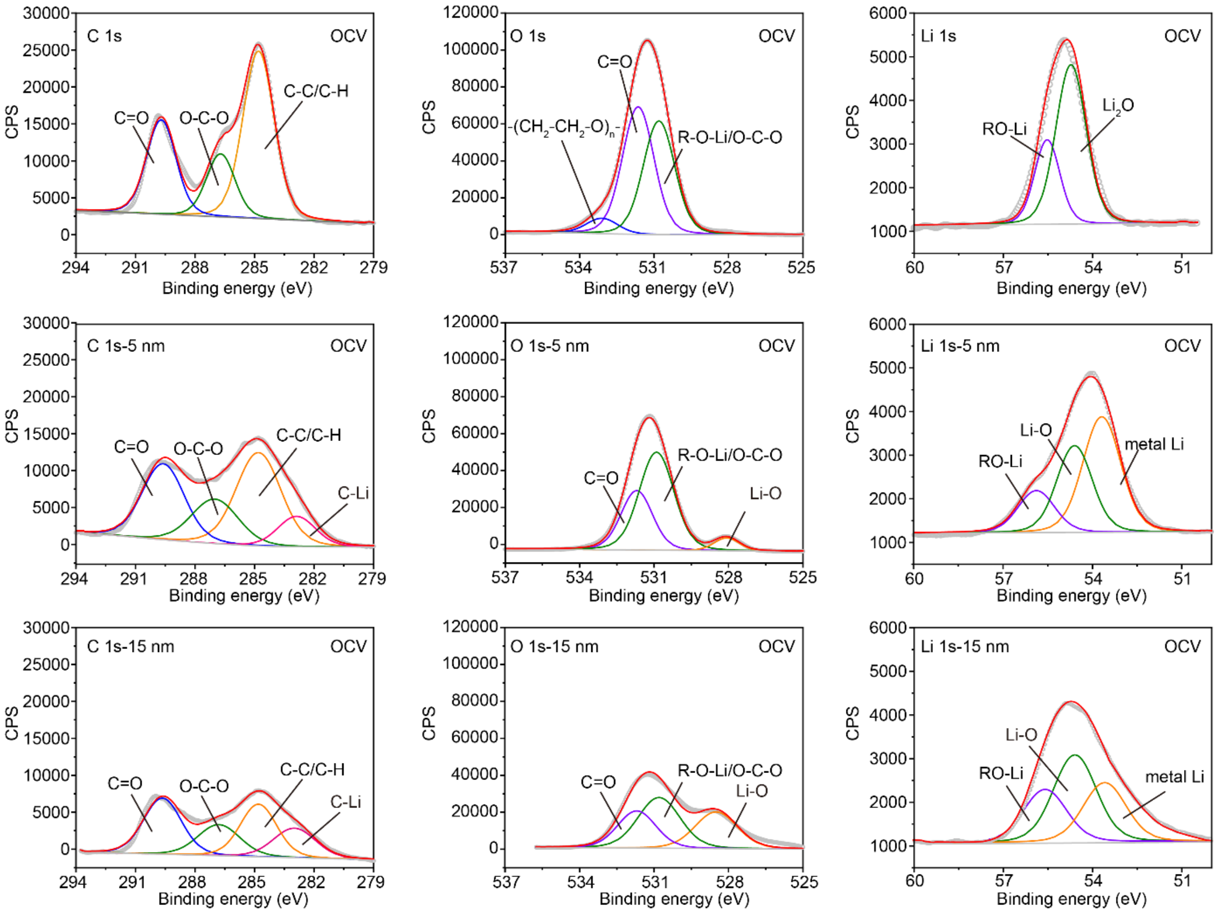

Figure S23. C 1s, O 1s, and Li 1s XPS depth profiles of the Li metal of Li/Li-Naph-LLZTO/Li at OCV.

Note: The Li anode was rinsed by anhydrous DME three times to remove residual Li-Naph(s) and dried under vacuum. The samples were transferred into the XPS test chamber in a sealed sample holder without exposure to an ambient atmosphere.

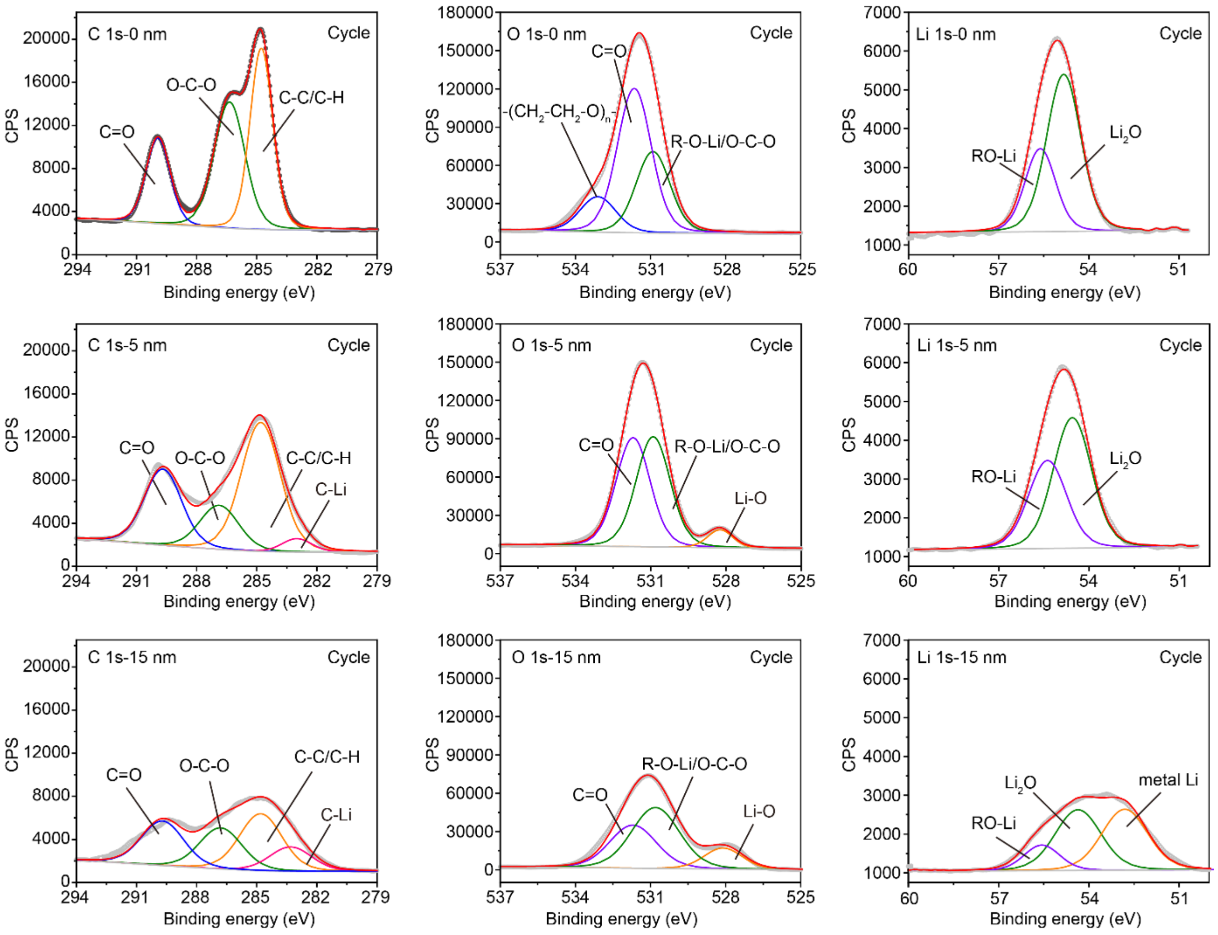

Figure S24. 1s, O 1s, and Li 1s XPS depth profiles of the Li metal of Li/Li-Naph-LLZTO/Li after cycling 20 h at 0.2 mA cm^{-2}.

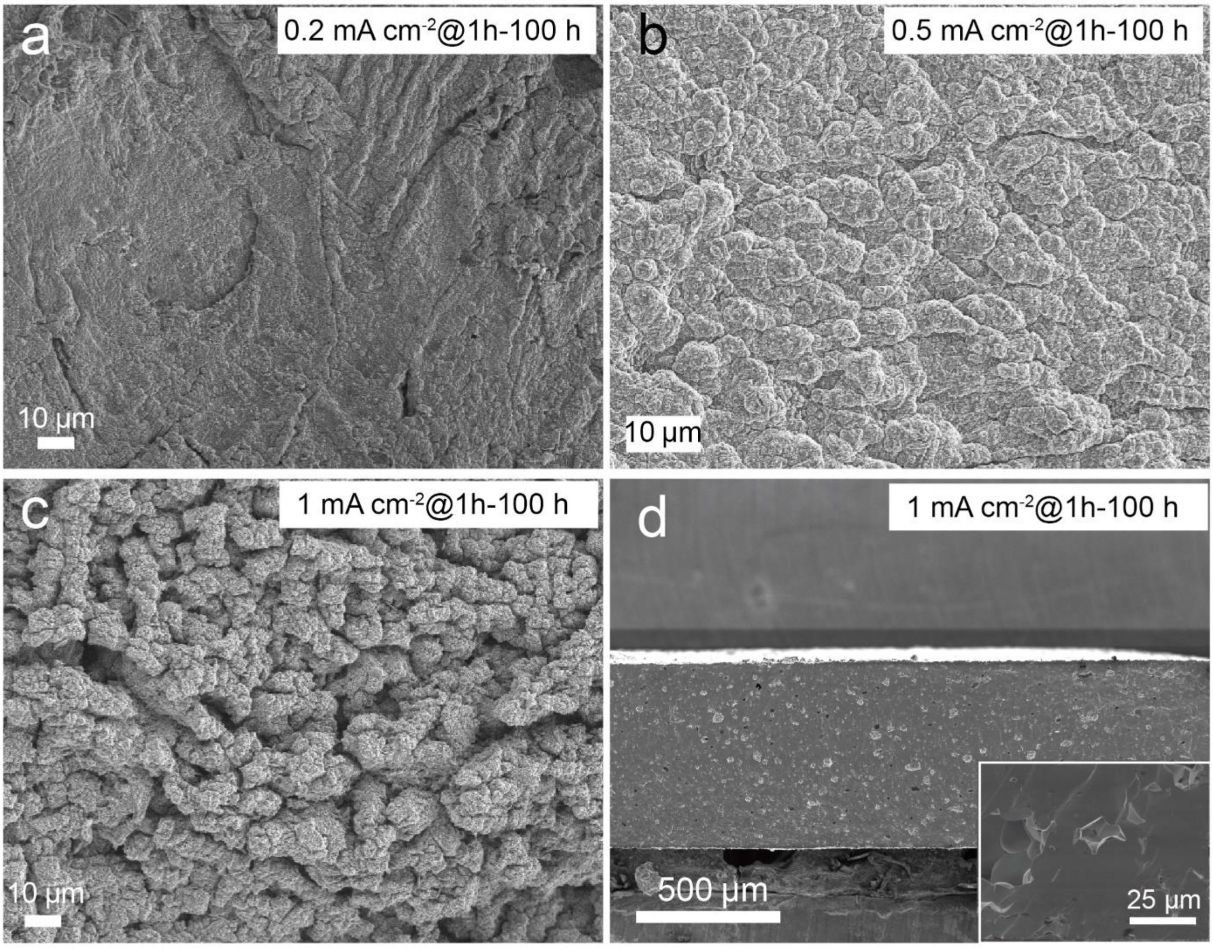

Figure S25. SEM images of Li metal of Li/Li-Naph-LLZTO/Li after cycling 100 h at (a) 0.2 mA cm^{-2}, (b) 0.5 mA cm^{-2} and (c) 1 mA cm^{-2}. (d) Cross-sectional SEM image of LLZTO pellet at 1 mA cm^{-2} after 100 h.

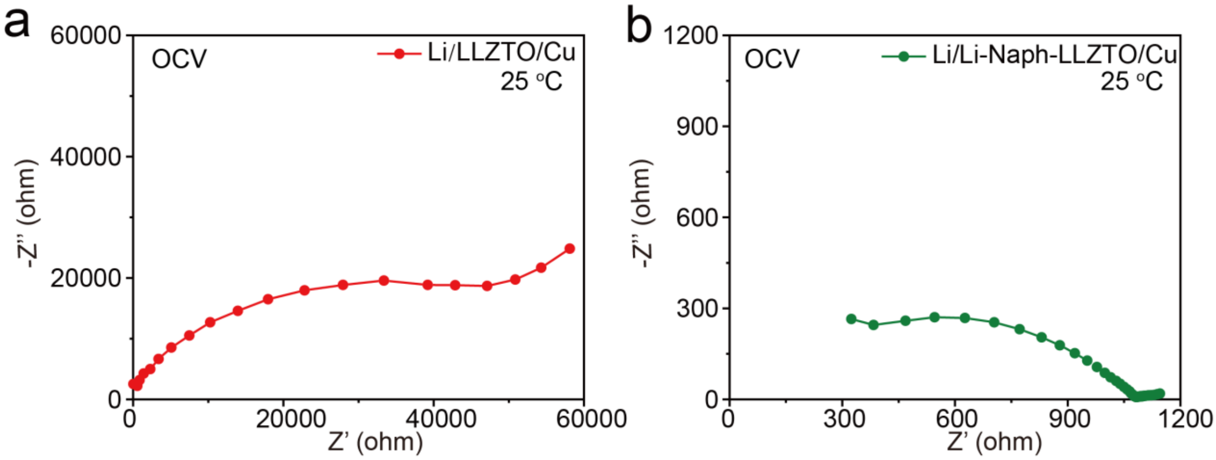

Figure S26. EIS measurements of (a) Li/ LLZTO/Cu and (b) Li/Li-Naph-LLZTO/Cu asymmetric cells.

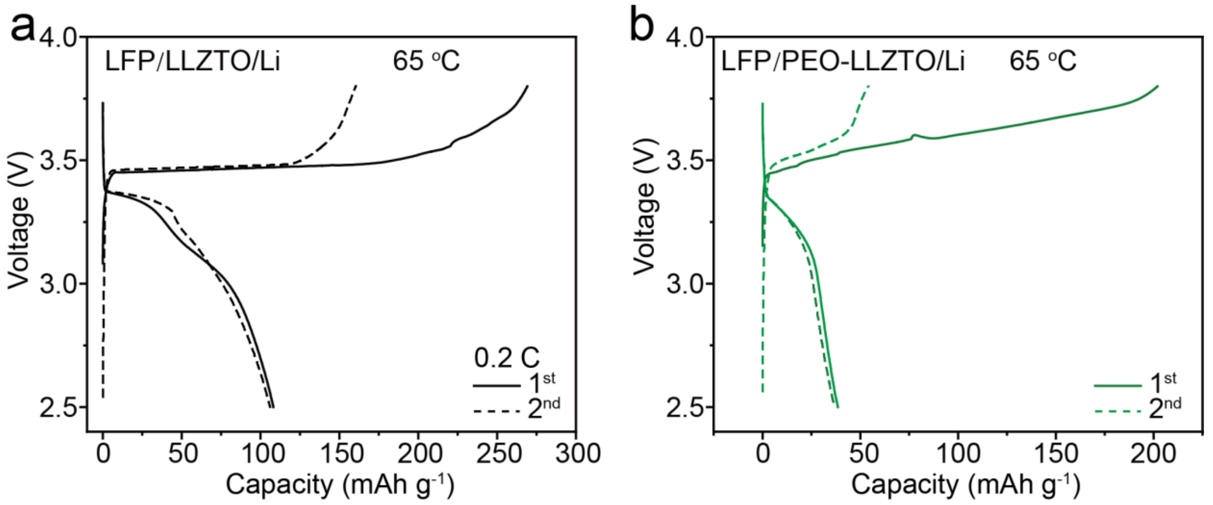

Figure S27. Galvanostatic charge and discharge voltage profiles at the current densities 0.2C of (a) the Li/LLZTO/LFP and (b) Li/PEO-LLZTO/LFP full cell.

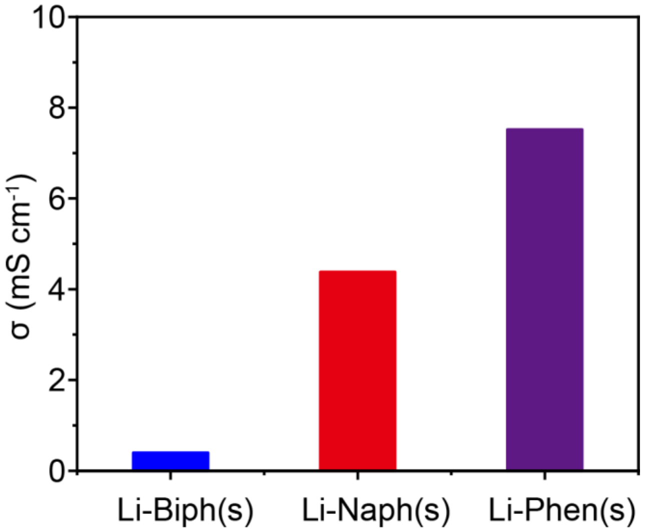

Figure S28. Ionic conductivity of Li-Phen(s), Li-Naph(s) and Li-Biph(s) at room temperature.

Note: To obtain the ionic conductivities of Li-Phen(s), Li-Naph(s), and Li-Biph(s), their solutions were cautiously dropped on the stainless-steel disc and vacuumed for 15 min to assemble symmetric cells with SS discs.

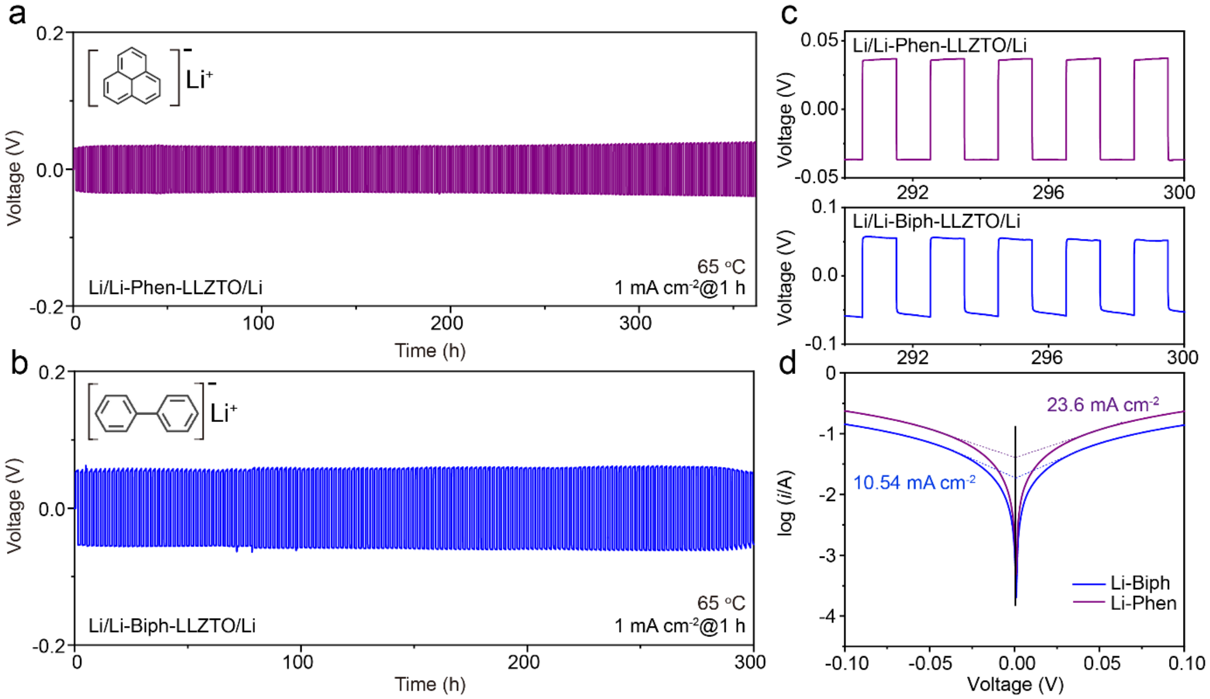

Figure S29. Galvanostatic cycling performance of the (a) Li/Li-Phen-LLZTO/Li and (b) Li/Li-Biph-LLZTO/Li symmetric cells at a current density of 1 mA cm^{-2} with a fixed areal capacity of 1 mAh cm^{-2}. (c) Voltage curves corresponding to different cycle stages of (a) and (b). (d) Exchange current densities of Li/Li-Biph/Li and Li/Li-Phen/Li.

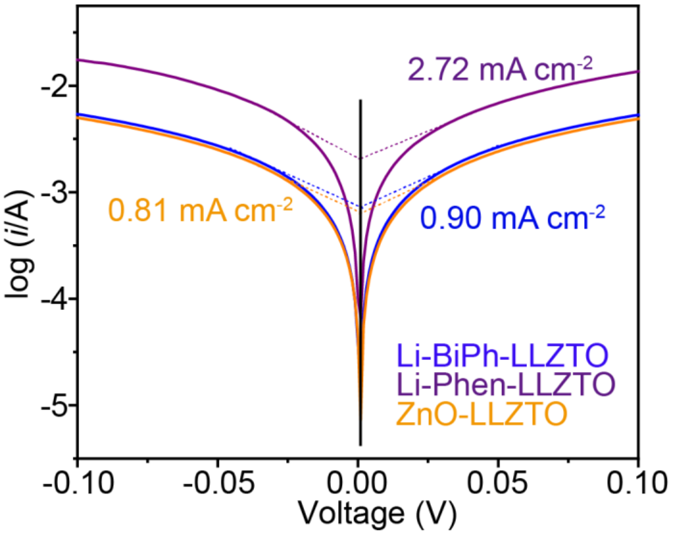

Figure S30. Exchange current measurements of Li-Biph(s)/LLZTO, Li-Phen(s)/LLZTO and ZnO-LLZTO.

Table S1. Results of conductivity measurements for Li-Naph(s).

R_0 (ohm)	15.3
R_1 (ohm)	17.4
R_2 (ohm)	26.5
R_i (ohm)	2.58
R_e (ohm)	11.2
σ_i (S cm^{-1})	4.38×10^{-3}
σ_e (S cm^{-1})	1.01×10^{-3}

Table S2. Electrochemical impedance and d.c. ASR for Li/Li-Naph-LLZTO/Li cells at 25 ^{o}C and 65 ^{o}C.

Li/Li-Naph-LLZTO/Li (Ω cm^2)	Bulk/high-frequency ASR	GB ASR	*Interfacial EIS ASR	Total EIS ASR	d.c. ASR	*Interfacial d.c. ASR
25 ^{o}C	94.6	148.8	131.8	507.0	740	129.9
65 ^{o}C	33.8	5.7	9.9	59.3	60	10.4

*Interfacial d.c. ASR is calculated by subtracting garnet ASR from d.c. ASR (Based on Ohm's law, the ASR was calculated from the stripping/plating test). The garnet ASR was obtained from the EIS garnet conductivity measurement of the Ag/LLZTO/Ag symmetric cells, and the total garnet ASR is 480.3 Ω cm^2 at 25 ^{o}C and 39.3 Ω cm^2 at 65 ^{o}C.

*Interfacial EIS ASR values were estimated based on the equivalent circuit in the insets of Fig. 2a and 2b, which shows a good fitting result.

Table S3. Area-specific resistances (R_{int}), corresponding capacitance values (C), characteristic time constants (τ), and CPE exponent (n) estimated from the impedance spectra of symmetric Li/LLZTO/Li or Li/Li-Naph-LLZTO/Li cells at 25 °C and 65 °C according to the equivalent circuit in insets of Figure 2a and 2b. Related parameters for interface charge transport process.

cell	R_{int} (Ω cm^2)	C (F cm^{-2})	τ	n
Li/LLZTO/Li (25 °C)	8399.5	1.2×10^{-8}	0.1 ms	0.71
Li/LLZTO/Li (65 °C)	483.4	1.4×10^{-8}	6.8 μs	0.74
Li/Li-Naph-LLZTO/Li (25 °C)	263.6	2.8×10^{-7}	75.0 μs	0.73
Li/Li-Naph-LLZTO/Li (65 °C)	19.8	2.5×10^{-7}	5.0 μs	0.72

$C = (R^{1-n}CPE)^{1/n}$

$\tau = RC$

Table S4 The relative density of LLZTO ceramic pellets evaluated by Archimedes' principle.

	m_{LLZTO}	$\rho_{ethanol}$	$m_{submerged}$	ρ_{real}	$\rho_{theoretical}$	$\rho_{relative}$
Group1	0.2715 g	0.789 g cm^{-3}	0.2311 g	5.302 g cm^{-3}	5.5 g cm^{-3}	96.4%
Group2	0.2699 g	0.789 g cm^{-3}	0.2297 g	5.297 g cm^{-3}	5.5 g cm^{-3}	96.3%

The relative density of LLZTO ceramic pellets was evaluated by the Archimedes' principle, which is shown as the following formula,

$\rho_{relative} = \rho_{real}/\rho_{theoretical}\times100\%$ (1)

$\rho_{real} = m_{LLZTO}\bullet\rho_{ethanol}/(m_{LLZTO} - m_{submerged})$ (2)

Where the $\rho_{relative}$ is the relative density of the LLZTO, the ρ_{real} and $\rho_{theoretical}$ are the tested density and the theoretical density of the LLZTO, m_{LLZTO} is the mass of the LLZTO, $m_{submerged}$ is the apparent mass of the LLZTO when submerged in the ethanol, and $\rho_{ethanol}$ is the density of the ethanol. Two groups of relative density data are shown in Table S4.

Table S5. Comparison of electrochemical performances between various interlayer modifications.

Interlayer material	Fabrication method	Test temp.	Interfacial resistance ($\Omega.cm^2$)	Cycling performance	Cycles (h)	CCD	Ref.
Li-Naph (s)	Cold process	25 °C<br>65 °C	130.8<br>9.9	0.2 mA cm^{-2}@1 h<br>1 mA cm^{-2}@1 h	1200<br>580	1.7 mA cm^{-2}	This work
Li_3N	Electron beam thermal deposition system	40 °C	142	0.1 mA cm^{-2} @5 min	210	/	*Nano Lett.* **2018,** 18, 7414. (Ref. 23)
Al_2O_3	Atomic layer deposition	25 °C	1	0.2 mA cm^{-2} @0.5 h	90	/	*Nat. Mater.* **2017,** 16, 572. (Ref. 24)
Si	Plasma-enhanced chemical vapor deposition	RT	127	0.2 mA cm^{-2}@10 min	30	0.2 mA cm^{-2}	*J. Am. Chem. Soc.* **2016,** 138, 12258. (Ref. 27)
Ge	Electron beam thermal deposition system	25 °C	115	0.05 mA cm^{-2}@10 min	150	/	*Adv. Mater.* **2017,** 29, 1606042. (Ref. 28)
Cu_6Sn_5	Magnetron sputtering and annealing	25 °C	236	0.25 mA cm^{-2}@0.5 h	300	/	*ACS Energy Letters* **2019,** 4, 1725. (Ref. 29)
Cu/Li_3N	Magnetron sputtering	25 °C	83.4	0.25 mA cm^{-2}@0.5 h	1000	1.2 mA cm^{-2}	*Energy Environ. Sci.* **2020,** 13, 127. (Ref. 35)
Mo/Li_2S	Polishing	100 °C	14	0.2 mA cm^{-2}@0.5 h	40	/	*Energy Environ. Sci.* **2019,** 12, 1404. (Ref. 36)
Li_ySn/Li_3N	Magnetron sputtering	25 °C	164.8	0.1 mA cm^{-2}@0.5 h	1200	1.5 mA cm^{-2}	*Angew. Chem. Int. Ed.* **2020,** 59, 11784. (Ref. 37)
C reduction	700 °C	65 °C	45	0.1 mA cm^{-2} @1 h<br>0.4 mA cm^{-2} @1 h	450<br>150	/	*J. Am. Chem. Soc.* **2018,** 140, 6448 (Ref. 50)
ZnO	Atomic layer deposition	RT	20	0.1 mA cm^{-2} @0.17 h	50	/	*Nano Lett.* **2017,** 17, 565. (Ref. 51)
Sn	250 °C	30 °C	46.6	0.5 mA cm^{-2}@0.5 h	500	0.5 mA cm^{-2}	*Adv. Energy Mater.* **2018,** 8, 1701963. (Ref. 52)

References

[50] Y. Li, X. Chen, A. Dolocan, Z. Cui, S. Xin, L. Xue, H. Xu, K. Park, J. B. Goodenough, *J. Am. Chem. Soc.* **2018,** 140, 6448.

[51] C. Wang, Y. Gong, B. Liu, K. Fu, Y. Yao, E. Hitz, Y. Li, J. Dai, S. Xu, W. Luo, E. D. Wachsman, L. Hu, *Nano Lett.* **2017,** 17, 565.

[52] C. Wang, H. Xie, L. Zhang, Y. Gong, G. Pastel, J. Dai, B. Liu, E. D. Wachsman, L. Hu, *Adv. Energy Mater*. **2018,** 8, 1701963.
